# An Efficient and Cohesive System for Enhanced Accuracy in Malignant Brain Tumor Diagnosis

**DOI:** 10.2174/0115734056447226260528100852

**Published:** 2026-06-10

**Authors:** Laouni Djafri, Yacine Gafour

**Affiliations:** 1 Department of Mathematics, Ibn Khaldoun University of Tiaret, Tiaret, Algeria; 2 LIM Laboratory of Informatics and Mathematics, Ibn Khaldoun University of Tiaret, Tiaret, Algeria

**Keywords:** Brain tumor, Deep learning, Ensemble learning, Segmentation, Features extraction, Tumor diagnosis

## Abstract

**Introduction/Objective:**

Brain tumors present a significant health challenge, making an accurate diagnosis crucial for effective treatment and improved patient outcomes. This study aims to develop SCSFE-DiagBT, an advanced deep learning framework that integrates classification and segmentation. It is designed for enhanced brain tumor diagnosis using MRI scans.

**Methods:**

We propose a Stacked Classification and Segmentation Model with Feature Extraction (SCSFE-DiagBT). This model unifies the processes of classification and segmentation into a single diagnostic pipeline. It leverages the complementarity of both tasks by employing a deep learning approach to efficiently analyze MRI scans.

**Results:**

Experimental results indicate substantial improvements in performance metrics. In classification, metrics such as accuracy, precision, and recall improved by up to 31% compared to baseline models, with high accuracy. For segmentation, the model achieved a 12% improvement in the Dice coefficient, reaching an accuracy of 99.58% and a Dice score of 86.30%. This demonstrates robust generalization and minimal overfitting.

**Discussion:**

The findings underscore the effectiveness of integrating classification and segmentation in the diagnosis of brain tumors. The enhanced interpretability through feature extraction further supports the model's utility in clinical settings, potentially reducing diagnostic variability associated with manual interpretations.

**Conclusion:**

SCSFE-DiagBT emerges as a highly accurate and viable tool for brain tumor diagnosis. It offers significant advancements over traditional methods and promising implications for improving patient care and outcomes in neuro-oncology.

## 1 INTRODUCTION

The brain, a highly intricate and vital organ, governs essential functions including movement, memory, emotions, and cognition. However, its complexity makes it susceptible to various disorders, with brain tumors presenting a particularly severe threat. These tumors arise from abnormal and uncontrolled cell proliferation, disrupting normal brain activity and leading to significant health complications or even death if not identified and treated promptly. Brain tumors are primarily classified into two categories: benign and malignant. Common forms include gliomas, meningiomas, and pituitary tumors, each differing in terms of location, behavior, and severity. Early and accurate diagnosis is crucial for improving treatment outcomes and survival rates. Medical imaging, particularly through magnetic resonance imaging (MRI), is essential for the detection and monitoring of brain tumors. However, the manual analysis of these images poses significant challenges for radiologists due to the increasing volume of medical data and subtle visual differences among tumor types, often resulting in delays or misdiagnoses, such as in multi-class tumor grading and lesion delineation [1]. To enhance the efficiency of model training, data preprocessing is imperative. This includes data augmentation techniques that artificially increase the quantity and diversity of training datasets through modifications such as horizontal flips, rotations, and random cropping [2]. Research has shown that optimizing convolutional neural networks (CNNs) with data augmentation and denoising techniques can significantly improve the generalizability and robustness of brain tumor detection systems [3]. Techniques such as CNNs, Vision Transformers (ViTs), and hybrid attention-based models have shown remarkable success in automating tumor detection, classification, and segmentation. These systems learn spatial and semantic representations directly from volumetric MRI data, achieving promising diagnostic performance in research contexts [4, 5]. For instance, hybrid CNN-Transformer models proposed by Goceri [6] achieved over 98% accuracy in glioma grading tasks. Multi-branch models that integrate MobileNet, ViT, and LSTM effectively capture spatial, semantic, and temporal features across MRI slices. While these models demonstrate high multiclass accuracy, they remain vulnerable to class imbalance and overfitting [7].

Despite these developments, there are still significant challenges in real-world clinical applications. These challenges include severe class imbalance across tumor categories, limited availability of well-annotated MRI datasets, variations in data acquisition protocols between institutions, and inadequate interpretability of deep learning models. [5, 8]. Recent studies have examined preprocessing strategies like skull stripping and contrast enhancement (*e.g*., CLAHE), as well as algorithmic solutions such as ensemble learning and explainability techniques like Grad-CAM and SHAP, aimed at enhancing model robustness and transparency [7-9]. Recent advance-ments in brain tumor classification focus on hybrid and ensemble-based deep learning systems that incorporate spatial encoding, attention mechanisms, and model interpretability. Notably, a CNN architecture augmented with Transformer blocks has shown exceptional performance in glioma grading, adeptly capturing local textures and global spatial dependencies while maintaining efficiency through global pooling and multi-loss optimization [6]. To enhance model transparency and robustness in brain tumor classification, explainable stacking ensemble models have been developed. These models combine EfficientNet, MobileNet, GoogleNet, and CapsuleNet with a CatBoost meta-classifier. These models, integrated with preprocessing techniques such as CLAHE and skull stripping, as well as dimensionality reduction through PCA and class balancing methods like Borderline-SMOTE, achieved F1-scores exceeding 97%. Additionally, Grad-CAM visualizations aid in clinical interpretability. However, the architectural complexity may pose challenges for real-world deployment [8]. A new model proposed by Anand *et al*. for classifying brain tumors in MRI images utilized a Weighted Average Ensemble Deep Learning approach [10]. This approach combines three pivotal components: a CNN without augmentation, a transfer learning-based model, and a CNN enhanced through data augmentation techniques. This innovative framework effectively merges distinct features using weighted averages optimized *via* grid search, significantly improving both accuracy and robustness. In a related work by Saeedi *et al*., six machine learning methods were compared alongside two deep learning networks to further enhance tumor detection accuracy [11]. Additionally, the Brain Tumor Segmentation and Classification Network (BTSCNet) was introduced to classify three types of brain tumors, highlighting the effectiveness of deep CNNs. This model incorporates a 26-layer architecture augmented with VGG16 and Xception, specifically trained with class-wise augmentation to address issues of class imbalance and overfitting [12].

Furthermore, segmentation techniques are just as important as classification in the diagnosis of brain tumors. It accurately defines the boundaries of the tumor within MRI images, enhancing the accuracy of diagnosis and treatment strategies. While classification categorizes tumors, segmentation provides detailed information necessary for effective clinical decision-making. In this context, innovative architectures like YOLO-TumorNet have emerged, transforming object detection frameworks into segmentation-capable models by integrating YOLOv10 with InceptionNeXt, BiFPN, and Multi-Scale Spatial Pyramid Attention (MSPA). These advancements enable multiscale feature extraction and boundary refinement, facilitating precise localization and delineation of tumors with complex morphologies [13]. Additionally, a bi-directional cascade Gaussian kernel GAN (BCK-GAN) was developed by Anjana *et al.*, combining generative feature modeling with adaptive optimization to address noise in MRI inputs and mitigate under-segmentation in low-contrast regions [14]. To address these needs, we propose an intelligent system employing deep learning that performs three primary tasks: classifying brain MRIs into four categories (glioma, meningioma, pituitary tumor, or healthy), segmenting the tumor area within the brain image for precise localization, and extracting morphological features of the tumor, including surface area, perimeter, width, height, and circularity. The work presented by Joy *et al*. utilizes a deep CNN architecture comprising multiple convolutional, pooling, and dense layers [15]. The authors employ the Adam optimizer together with categorical cross-entropy loss for training, and they also apply data augmentation techniques. This approach leads to a high training accuracy of 99% and a validation accuracy of 91.5%. We also find the work suggested by Pani *et al*. to be equally important as the previous studies [16]. They propose a novel approach to brain tumor segmentation using synthetic T1CE scans generated from T1 scans through an image-to-image translation framework called VarVit-GAN. Additionally, they implement an adaptive random patch selection algorithm (ARPS) to address issues related to data scarcity and sparsity. Asadi *et al*. introduced the StyleGAN2-ada model for generating synthetic brain images [17]. They employed U-Nets for automatic glioma segmentation and assessed the impact of synthetic data augmentation on U-Net performance, using the Dice coefficient as the primary evaluation metric. Wang *et al*. proposed a novel data augmentation technique called TensorMixup [18]. This technique combines image patches containing tumor regions from the MRI images of two randomly selected patients using a tensor. The resulting mixed image patches are used to train a 3D U-Net architecture for brain tumor segmentation.

**Fig. (1):**
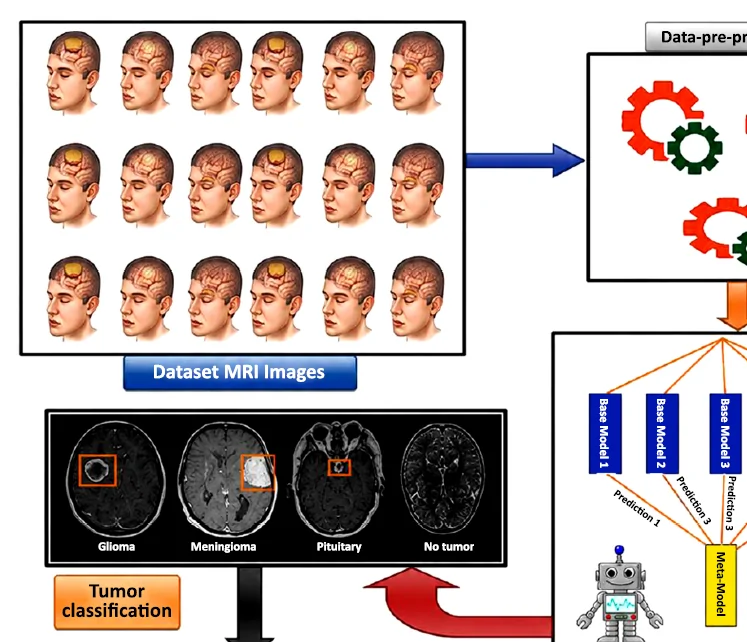
Process for the accurate diagnosis of malignant brain tumors.

Figure **1** illustrates the operating principle of the accurate diagnosis process for malignant brain tumors proposed in this work.

## 2 RELATED WORK

Accurate classification and segmentation of brain tumors are essential for reaching reliable diagnoses. Traditionally, this field has relied on conventional techniques such as magnetic resonance imaging (MRI), biopsy, electroencephalography (EEG), and fluorescence imaging. Although these methods provide crucial insights into the presence and nature of tumors, they also have some limitations. To overcome these limitations, AI-based methods, particularly the integration of data processing with deep learning techniques, are increasingly being applied to achieve highly accurate diagnoses. Within this context, data augmentation plays a key role, standing out as a critical technique in medical imaging, especially for enhancing brain tumor classification and segmentation. It works by generating variations of existing training images to expand dataset diversity, thereby improving the performance and robustness of deep learning models. This process enriches both the size and heterogeneity of the training data through operations such as horizontal flipping, rotation, resizing, and random cropping [2]. In this domain, Zuo *et al*. proposed the Multi-Channel Fusion Diffusion Model (MCFDiffusion), specifically designed to augment brain tumor MRI datasets and thereby improve the performance of image classification and segmentation models [19]. Similarly, Alanazi employed a comprehensive feature engineering strategy that integrates data augmentation techniques to enhance the accuracy of brain tumor classification [20]. In this work, the original MRI images were preprocessed by resizing them to a standardized input resolution of 224×224 with three color channels, followed by pixel value normalization within the range of 0 to 1. Moreover, another study proposed AGGrGAN, an ensemble of three GANs (two DCGAN variants and one WGAN) combined with style transfer to generate synthetic brain MRI scans [21]. In recent research, the authors proposed a hybrid brain-tumor classification framework that combines a Swin Transformer backbone with AE-cGAN augmentation [22]. The Swin Transformer captures both local and global dependencies, while AE-cGAN generates diverse synthetic MR images to mitigate class imbalance and enrich feature representations. Together, these contributions demonstrate the effectiveness of combining transformer-based feature extraction with GAN-driven data augmentation for robust medical-image classification.

In addition to data augmentation, data balancing is equally critical for developing robust and reliable brain tumor classification systems. Class imbalance, which often results from the underrepresentation of specific tumor subtypes, can lead to significant bias during model training, reduce the algorithms' ability to generalize, and ultimately lead to high rates of misclassification. Therefore, addressing this problem is essential to ensure fair and accurate diagnostic predictions. In this context, Roy *et al*. proposed DeepEFE, an interpretable assembly pipeline that combines a Dual-GAN for generating high-quality synthetic minority-class MRI samples with multiple feature-extraction techniques to tackle severe class imbalance [23]. Along similar lines, Deepak and P. M. Ameer investigated class imbalance in deep learning-based brain tumor classification from MRI data, proposing a novel class-weighted focal loss to improve feature learning in CNN models [24. Moreover, Duan *et al*. introduced a pseudo-labeling approach tailored to medical imaging, incorporating a pseudo-label filter that performs image-level screening based on uncertainty estimates [25]. Their proposed IPL-Net framework integrates this imbalanced pseudo-labeling method with an improved version of Big-nnU-Net while leveraging a specialized loss function designed for segmentation tasks characterized by foreground-background imbalance. This approach significantly enhances overall segmentation performance.

Once the increased data has been processed and balanced, the next step involves training classification models capable of leveraging the improved datasets to maximize diagnostic accuracy in brain tumor analysis. Among the available methods, convolutional neural networks (CNNs) consistently stand out as the most effective for classifying medical images. In the context of brain tumor classification, well-established architectures such as Xception, Inception, ResNet, and VGG have demonstrated outstanding performance [26]. The Xception model, a highly efficient deep learning architecture, is particularly suited for tasks that require both high accuracy and computational efficiency. It can be adapted for multi-class classification and segmentation problems [27, 28]. The Inception architecture, known for its ability to process images efficiently, extracts salient features through the use of multiple convolutional filter sizes, and its performance can be further enhanced *via* transfer learning and fine-tuning [29]. ResNet, with its innovative residual learning mechanism, effectively mitigates the vanishing gradient problem, enabling the training of very deep networks [30, 31]. The VGG architecture, although comparatively simpler, has achieved remarkable success in image classification challenges such as the ImageNet Large Scale Visual Recognition Challenge (ILSVRC), proving its robustness and effectiveness [32].

Building on these foundations, recent studies propose advanced CNN-based architectures to further improve brain tumor classification. Kumar *et al*. developed a brain tumor classification framework using the VGG19 architecture trained on 7,023 MRI images from a Kaggle dataset [33]. Comprehensive preprocessing and data augmentation were applied to enhance feature representation and improve generalization capability to enhance transparency and interpretability in the decision-making process. Similarly, Kantu *et al*. developed the Conv-Attention Vision Transformer (CAViT), which integrates local and global attention mechanisms into ResNet-50 and VGG-19, achieving notable improvements in ROCAUC and F1-scores [34]. In parallel, lightweight architectures such as MobileNetV3 have demonstrated strong diagnostic accuracy with substantially lower computational requirements, making them particularly well-suited for multi-class brain tumor classification in resource-constrained clinical environments. Moreover, other advanced approaches include the use of DenseNet121 with transfer learning, which achieved an average classification accuracy of 96.90% in multi-class brain tumor detection [35], and the BTDN model, a ConvNet designed for automatic tumor recognition in MRI scans, which offers efficient resource utilization, enhanced grayscale MRI processing, and improved interpretability [36]. More recently, SparseSwinMDT, proposed by Li *et al*., integrates a sparse token representation mechanism with a Multipath Decision Tree classifier, delivering high accuracy and computational efficiency [37]. Furthermore, Panigrahi *et al*. introduced an ensemble approach that combines CNN pre-trained models with additional feature extraction layers and a majority voting scheme, achieving state-of-the-art accuracy of 99.34% on the Brain MRI Images for Brain Tumor Detection dataset [38].

Accurate classification establishes the foundation of precision medicine in neuro-oncology, guiding treatment strategies and supporting reliable predictions. However, classification alone is not enough. Once the type of tumor is identified, image segmentation becomes critically important, as it focuses on accurately locating the tumor and separating it from the surrounding healthy tissue in medical images. This step is essential in clinical practice, as accurate detection of tumor boundaries ensures effective surgical planning and precise targeting of radiation therapy. Several advanced segmentation methods have been proposed to meet these needs. For instance, PA-ResNet50 introduces an explainable panoptic segmentation framework that combines instance and semantic segmentation to achieve detailed tumor boundary delineation while managing the uncertainties inherent in brain imaging [39]. Building on this foundation, Cao *et al*. developed BSAU-Net, a novel brain tumor segmentation model designed to enhance performance through integrating attention mechanisms and edge feature extraction [40]. The model incorporates multiple modules, including input, down-sampling, bottom, and up-sampling layers, as well as spatial attention (SPA) and edge-aware (EA) modules. Leveraging a U-Net-based architecture, BSAU-Net employs both edge-aware and spatial attention mechanisms, which are further refined by a Conditional Random Field (CRF) model for precise boundary detection. Similarly, Zhang *et al*. introduced GDacFormer, a network for MRI brain tumor segmentation that integrates adversarial learning with an enhanced Transformer module known as DacFormer [41]. This module features multi-scale dilated attention and a novel next convolutional block (NCB) incorporating Transformer architecture, optimizing multi-scale feature extraction and improving segmentation accuracy. In another study, Wen *et al*. proposed FedHG, an algorithm designed to address challenges associated with limited datasets [42]. FedHG enhances model robustness by integrating Virtual Adversarial Training (VAT) into a 3D U-Net architecture and employs a federated model aggregation strategy that generates effective and balanced weights for seamless integration across distributed datasets. Furthermore, Renugadevi *et al*. presented a hybrid framework that combines pre-trained CNNs (ResNet50, VGG16, Xception, and DenseNet121) with Vision Transformer (ViT) modules within a U-Net-based design [43]. To expand the dataset, augmentation techniques such as rotation, scaling, and flipping were applied. The proposed HVU architecture follows an encoder–decoder structure, in which the encoder compresses the input images, and the decoder restores spatial resolution, enhanced by ViT components to improve global feature representation. The HVU-ED segmentation model, trained using the Adam optimizer with a learning rate of 0.001 for 50 epochs, achieved remarkable performance, with an accuracy of 98.91% and precision, recall, sensitivity, and specificity all reaching 98%.

## 3 MATERIALS AND METHODS

**Fig. (2):**
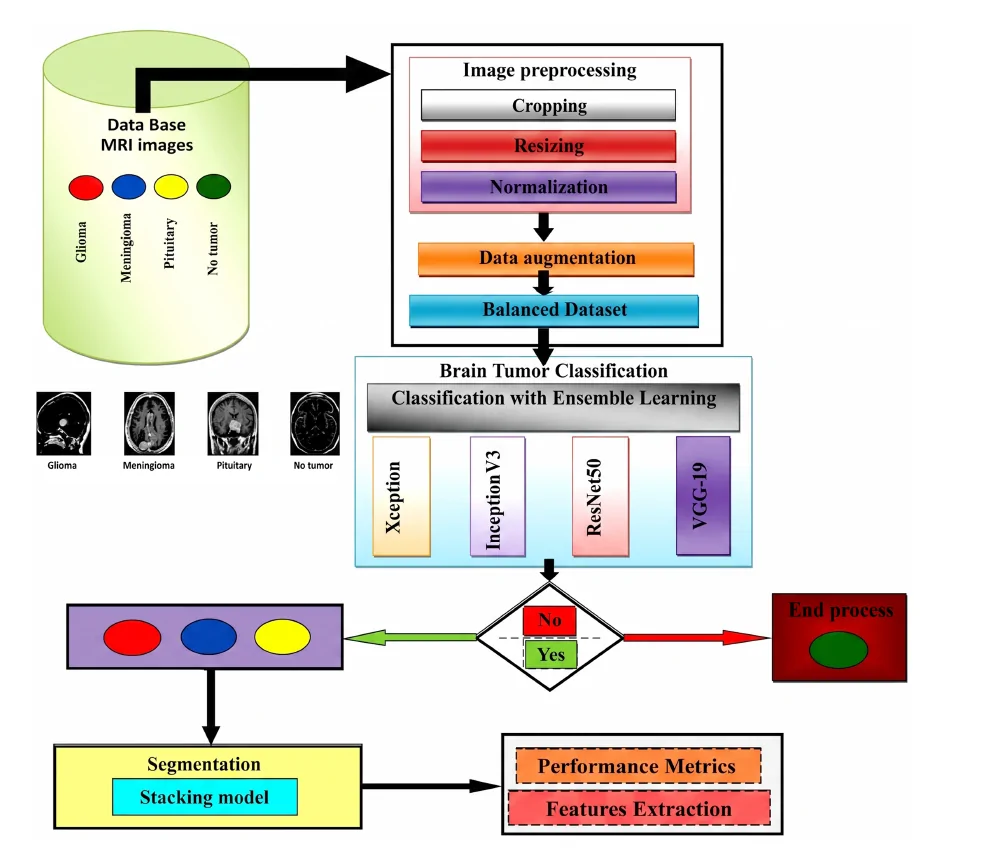
Proposed SCSFE-DiagBT system.

Developing an effective system for diagnosing brain tumors requires a structured and coherent architectural framework that integrates preprocessing of data, model selection, and implementation strategies. This section outlines the design and composition of our proposed system, detailing the key components involved in the classification and segmentation processes. We begin by describing the overall structure of our system, followed by the preprocessing techniques used on MRI images, including normalization, rescaling, and data augmentation. Next, we discuss our rationale for selecting specific deep learning architectures for classification and segmentation, highlighting their advantages in medical image analysis. We then examine the dataset structure, its origin, and the approach for partitioning the data into training, validation, and test sets. Finally, we demonstrate how to implement the classification and segmentation models, comparing and analyzing their performance metrics to illustrate how our system performs in real-world scenarios. Figure **2** shows our proposed system architecture:

### 3.1 Data Collection

The dataset used in our work was obtained from the Kaggle repository^1^ and comprises magnetic resonance imaging (MRI) scans categorized into four classes: Glioma, Meningioma, Pituitary Tumor, and No Tumor. In total, the dataset contains 7028 images, with 5721 images allocated for training and 1307 images reserved for testing. The class distribution, however, is imbalanced, with 1330 images of glioma, 1339 of meningioma, 1457 of pituitary tumors, and 1595 of no tumor cases. Such an imbalance can negatively impact model performance by biasing the classifier toward majority classes and reducing generalization ability. To address this limitation, a data augmentation strategy was applied, expanding each class to 2000 images, thereby generating a balanced training set of 8000 images. This preprocessing step ensured a more uniform class distribution and enhanced the robustness of the proposed system. Following augmentation, the dataset was partitioned into 80% for training and 20% for testing. The augmentation process was performed solely on the training data in order to avoid data leakage and to maintain an unbiased and reliable evaluation of the model’s performance.

### 3.2 Image Preprocessing

**Fig. (3):**
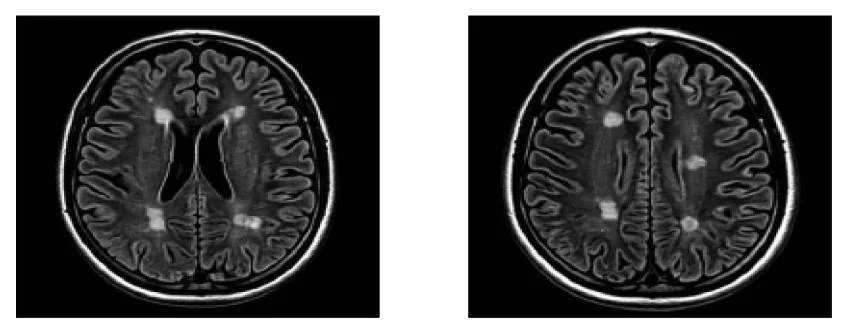
Cropping and resizing an image.

The pre-processing phase began with image preparation, ensuring the standardization of all MRI scans for subsequent model training. Specifically, each image was cropped and resized (Fig. **3**) to maintain consistent input dimensions across the dataset. To further optimize computational efficiency and stabilize the training process, pixel intensity values were normalized to a fixed range, thereby reducing variability caused by differences in image acquisition and improving model convergence.

In the next stage, data augmentation was applied to address overfitting, improve model generalization, and enhance robustness by introducing controlled variability into the training set. This technique, widely adopted in deep convolutional neural network (CNN) training, expands the dataset artificially through transformations such as shifting, rotation, flipping, cropping, and noise injection. However, in the context of medical imaging, particularly brain tumor detection, data augmentation must be applied with caution, as MRI scans contain highly sensitive anatomical structures. Inappropriate or excessive transformations may distort tumor morphology, leading to unreliable classification outcomes. To preserve diagnostic integrity, we implemented carefully constrained transformations, including slight rotations (≤10°), width and height shifts (≤0.1), moderate zooming (≤10%), controlled brightness adjustments (0.8–1.2), and horizontal flipping to generate mirrored representations. Conversely, extreme transformations were deliberately avoided, such as large rotations (±90° or more), aggressive zooming (>15%), or excessive brightness alterations (≥±30%), as these could introduce unrealistic distortions, obscure critical tumor regions, or compromise the model’s interpretability.

**Fig. (4):**
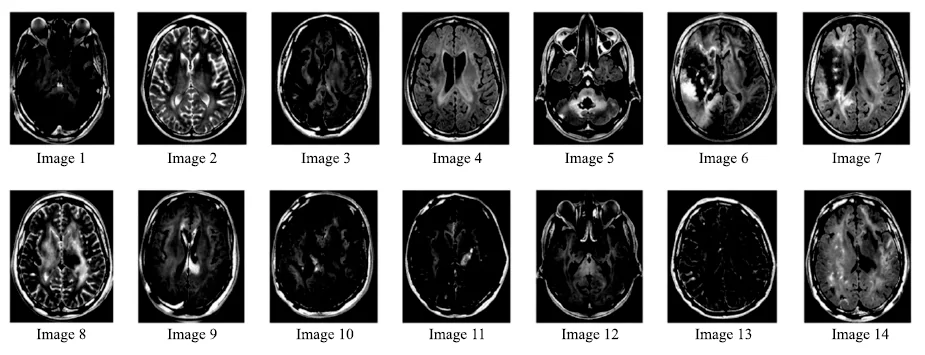
Glioma tumor using data augmentation.

**Fig. (5):**
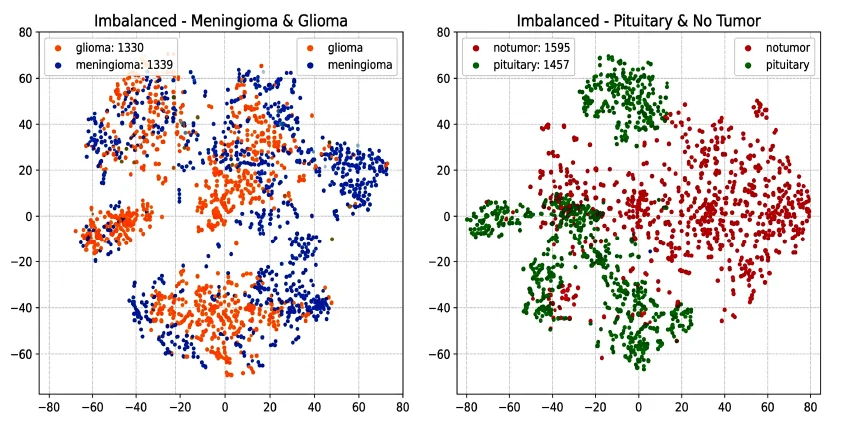
Data visualization of the classification dataset before balancing (imbalanced).

Due to the imbalance in image distribution across tumor classes, data augmentation was applied to enrich and balance the dataset, thereby improving the model's generalization ability. After this step, the dataset was divided into training, validation, and testing subsets to support systematic model development and reliable performance evaluation. Figures **4** and **5** show the class distribution before and after augmentation and balancing, confirming the effectiveness of this process in addressing class imbalance.

**Fig. (6):**
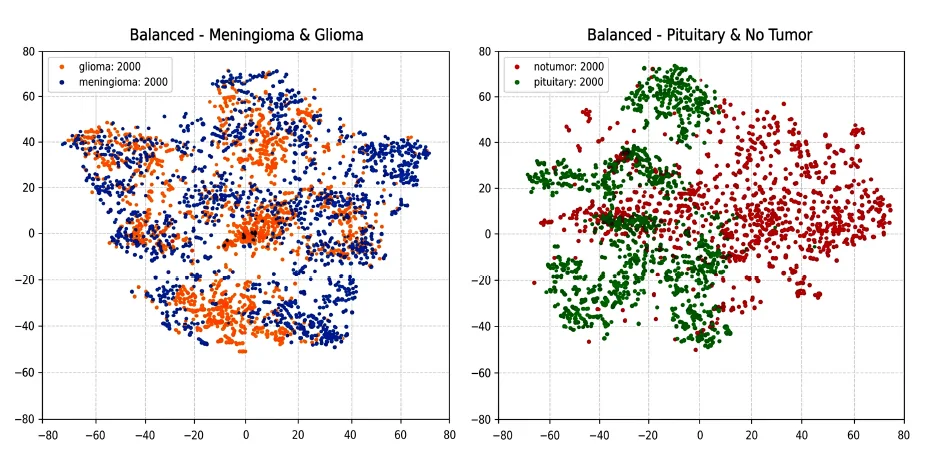
Data visualization of the classification dataset after balancing (balanced).

Figures **5** and **6** present the t-SNE (t-distributed Stochastic Neighbor Embedding) visualizations of the training dataset, highlighting the distribution of samples before and after the application of class-balancing techniques for brain tumor classification. These visualizations demonstrate how balancing reduces class overlap and improves the separability of tumor categories, thereby facilitating more effective model training. Following dataset preprocessing, the proposed system was structured into three core components: classification, segmentation, and feature extraction. This modular design ensures a comprehensive analysis pipeline, enabling not only the accurate identification of tumor types but also their precise localization and the extraction of discriminative features essential for robust diagnostic performance.

## 4 RESULTS AND DISCUSSION

### 4.1 Classification Models

In our proposed work, we selected four state-of-the-art classification models: Xception, InceptionV3, VGG-19, and ResNet-50, based on two key criteria. The first was architectural diversity, as each model introduces a distinct structural design and feature extraction mechanism. Xception leverages depthwise separable convolutions for efficient feature mapping. InceptionV3 employs multi-scale convolutional filters to capture patterns at different resolutions, VGG-19 utilizes deep sequential convolutional layers for hierarchical feature extraction, and ResNet-50 incorporates residual connections to effectively train very deep networks while addressing the vanishing gradient problem. This diversity enables the models to extract complementary features from MRI images, thereby enriching the overall representation space. By combining them through ensemble learning, the system benefits from the unique strengths of each model, leading to a more robust and comprehensive classification framework. The second criterion was proven performance in large-scale image recognition tasks, as all four architectures consistently demonstrate competitive results on benchmarks such as ImageNet, confirming their ability to generalize effectively across diverse datasets.

Once the models were selected, the next step was to determine the most appropriate ensemble strategy. Boosting, although capable of emphasizing difficult samples, often risks overfitting, which is a critical limitation in medical imaging, where misclassification may have severe clinical consequences. Bagging, typically effective with high-variance models such as decision trees, was considered less suitable because pre-trained deep learning models are already optimized and exhibit relatively low variance. Instead, we adopted stacking, which is particularly well-suited for leveraging the complementary capabilities of heterogeneous architectures. By employing a meta-learner to integrate predictions from the base models, stacking ensures that the final decision is informed by the most salient features extracted across models. This strategy not only enhances classification accuracy but also improves robustness, making it an ideal choice for brain tumor diagnosis.

There are several deep learning models for brain tumor classification, but relying on a single architecture may limit performance. To overcome this, we adopted an ensemble learning strategy, specifically a stacking-based approach, to leverage the complementary strengths of multiple models and enhance overall predictive accuracy.

The proposed stacking framework, as illustrated in Fig. (**7**), was implemented with carefully selected hyperparameters to ensure stable and efficient training. The base models Xception, InceptionV3, ResNet50, and VGG19 were optimized using the Adamax optimizer with a learning rate of 0.001, while categorical cross-entropy was employed as the loss function for the multi-class classification task with one-hot encoded labels. Each base model generated softmax probability distributions over the target classes, and these probability vectors were concatenated to form a unified meta-feature representation. For second-level integration, a lightweight fully connected neural network was used as the meta-learner, consisting of a single hidden layer with 32 neurons, ReLU activation, and a dropout rate of 0.3, followed by a softmax output layer. The meta-learner was trained using categorical cross-entropy and optimized with Adam (learning rate = 1 × 10^−4^). During stacking, the base model parameters were frozen to prevent information leakage, ensuring that only the meta-learner weights were updated. By operating on concatenated prediction probabilities rather than internal feature embeddings, the ensemble performs decision-level fusion, enabling the meta-learner to model non-linear relationships among base predictions while maintaining computational efficiency, robustness, and reproducibility.

The training strategy was divided into two stages. Initially, the base models were frozen, and only the upper layers were trained for 10 epochs to allow the network to capture high-level features. Subsequently, the deeper layers were unfrozen, and training continued for an additional 10 epochs, resulting in a total of 20 epochs. This fine-tuning phase enhanced the ability of the models to extract discriminative patterns, thereby improving generalization on unseen data. To further stabilize learning, we integrated a learning rate scheduler (ReduceLROnPlateau) that dynamically decreased the learning rate when the validation loss plateaued. Early stopping was also applied, with a patience of five epochs, to prevent overfitting and reduce training time. This method restored the best-performing weights, ensuring optimal generalization. In addition, dropout was introduced twice within the fully connected layers to mitigate overfitting by randomly deactivating neurons during training. After classification, cases identified as tumor-positive were directed to the segmentation module, where the ResUNet model was used to accurately locate and delineate tumor regions.

Below, we present the statistical evaluation and performance metrics of the classification models, analyzed individually and as part of the proposed SCSFE-DiagBT system.

In the first experiment, we evaluated the performance of the Xception model separately. The classification results are summarized in Table **1** and illustrated in Fig. (**8**).

From Table **1**, it can be observed that the Xception model achieves an accuracy of 99.22% at epoch 14, which remains stable until epoch 18. The loss reaches 2.77% at epoch 13 and remains constant up to epoch 18. Similarly, the precision attains a maximum value of 99.57%, while the recall, validation accuracy, validation precision, and validation recall all reach a peak value of 99.93%, reflecting strong overall classification performance.

As shown in Fig. (**8**), the training accuracy and validation accuracy exhibit a synchronous growth relationship, similar to the behavior of training and validation loss. Importantly, no signs of overfitting are observed, as the validation loss does not increase while the training loss continues to decrease. This indicates that the model is not merely memorizing the training data but is learning meaningful representations. Furthermore, the simultaneous decrease in both training and validation loss suggests that the model demonstrates good generalization ability, maintaining strong performance on unseen data.

In the second experiment, we evaluated the performance of the InceptionV3 model separately. The classification results are summarized in Table **2** and illustrated in Fig. (**9**).

From Table **2**, it can be observed that the model achieves an accuracy of 99.03% at epoch 14, with a gradual improvement until epoch 20, where accuracy reaches 99.20%, and the loss decreases to approximately 2.5%. Precision follows a similar trend, starting at 99.14% at epoch 14 and increasing to a peak of 99.91% by epoch 20. Furthermore, recall, validation accuracy, and validation precision converge to 99.97%, highlighting the model’s ability to maintain consistent and reliable predictions across all classes.

From Fig. (**9**), it is evident that training accuracy and validation accuracy remain strongly correlated throughout the training process, with only minor fluctuations observed at epochs 4 and 16. Likewise, training and validation losses follow a proportional downward trend, with a slight deviation at epoch 16. The close alignment between training and validation curves provides clear evidence of the model’s stability, indicating the absence of overfitting. This behavior confirms that the model not only learns effectively from the training data but also demonstrates strong generalization. In the third experiment, we conducted an independent evaluation of the ResNet-50 model to assess its classification performance. The results are presented in Table **3** and visualized in Fig. (**10**).

From Table **3**, we observe that the ResNet-50 model achieves lower accuracy compared to the two previously evaluated models. Its maximum training accuracy reaches 94.39%, but this is accompanied by a relatively high loss of 15.5%. Validation accuracy also shows considerable instability, peaking at 89.27% at epoch 14 before dropping to 83.16% at epoch 20. This inconsistency suggests that the model struggles to maintain stable performance during training. A similar trend is observed in the validation recall, which reaches 82.52% at epoch 19 but decreases to 80.74% at epoch 20. Such dynamics strongly indicate high variance and overfitting. Given that the learning rate remained stable (0.001, reduced only at epoch 20) and the optimizer was consistent, the instability is not due to the optimization settings. Instead, it is attributed to the depth and representational capacity of ResNet-50. The reason lies in the mismatch between the model’s effective complexity and the dataset’s size and variability.

From Fig. (**10**), the training and validation accuracy curves display pronounced fluctuations around epochs 16 and 18. This pattern is a clear indication of overfitting, where the model continues to optimize on the training set but fails to sustain accuracy on the validation data. This overfitting significantly undermines the model's ability to generalize, especially when compared to the more stable and reliable performance of Xception and InceptionV3.

In the fourth experiment, we independently evaluated the performance of the VGG-19 model to analyze its effectiveness in brain tumor classification. The corresponding results are summarized in Table **4** and illustrated in Fig. (**11**), providing detailed insights into the model’s training and validation behavior across different epochs.

From Table **4**, we observe a marked decline in performance compared to the previously tested models. The VGG-19 model achieves a maximum training accuracy of only 74.71%, accompanied by a notably high loss of 62.49%. Precision and recall also remain comparatively low, at 79.23% and 68.46%, respectively, reflecting the model’s limited ability to correctly identify and classify tumor types.

Validation metrics further highlight the instability of the model. Validation accuracy reaches 79.63% at epoch 14 but shows fluctuations, dropping to 78.71% in epochs 17–19 before stabilizing again at 79.63% by epoch 20. Validation precision demonstrates an even clearer downward trend, starting at 89.87% in the first epoch, decreasing to 85.23% by epoch 8, and falling further to 83.73% at epoch 20. Similarly, validation recall exhibits instability, with noticeable fluctuations around epochs 8 and 14, before peaking at 74.88% in the final epoch. This consistent decrease in validation performance despite improved training metrics demonstrates classic overfitting behavior. Unlike ResNet-50, VGG-19 does not show sudden jumps but rather a steady widening gap between training and validation curves. The validation loss also remains relatively high and fluctuating, confirming limited generalization capability. Architecturally, VGG-19 contains a very large number of parameters due to its deep convolutional stacks and fully connected layers, yet it lacks modern efficiency mechanisms such as residual connections or multi-scale feature extraction. This makes it both computationally heavy and less adaptable to subtle tumor morphology variations.

Importantly, both models were trained under identical preprocessing pipelines, learning rates (0.001), and optimization strategies. Since other architectures trained under the same conditions achieved more stable validation results, the performance discrepancy cannot be primarily attributed to initialization or optimizer configuration. Instead, it reflects architectural sensitivity to dataset scale, class distribution, and feature complexity.

From Fig. (**11**), the training and validation accuracy curves generally follow a similar trend, but isolated divergences at epochs 3 and 18 indicate instability in the learning process. A comparable pattern is observed for training and validation loss, where proportional decreases are occasionally disrupted by irregular spikes, particularly at epoch 12. These irregularities suggest that the VGG-19 model struggles with generalization and is more prone to overfitting compared to Xception, InceptionV3, and ResNet-50.

Additionally, we used other evaluation metrics, including the ROC/AUC curve and confusion matrix, to further confirm the classification performance of each model.

**Fig. (12):**
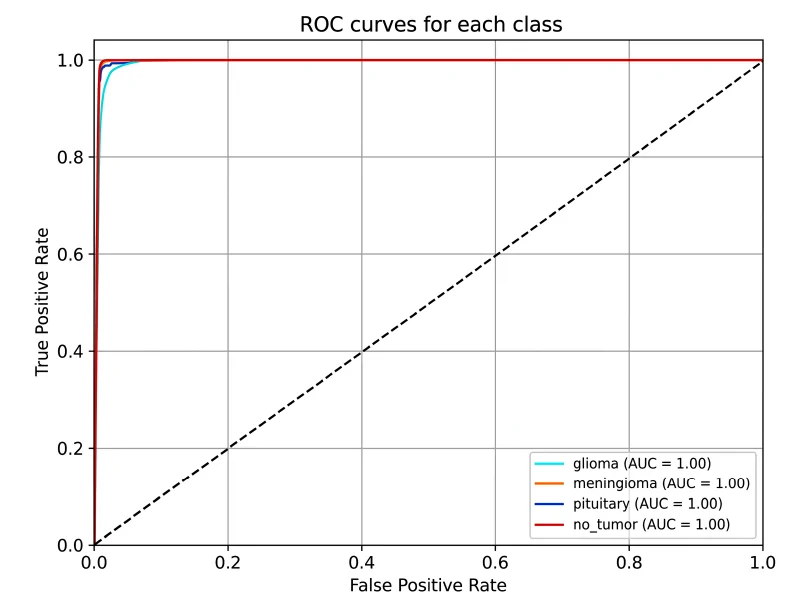
Roc/Auc curve for Xception.

**Fig. (13):**
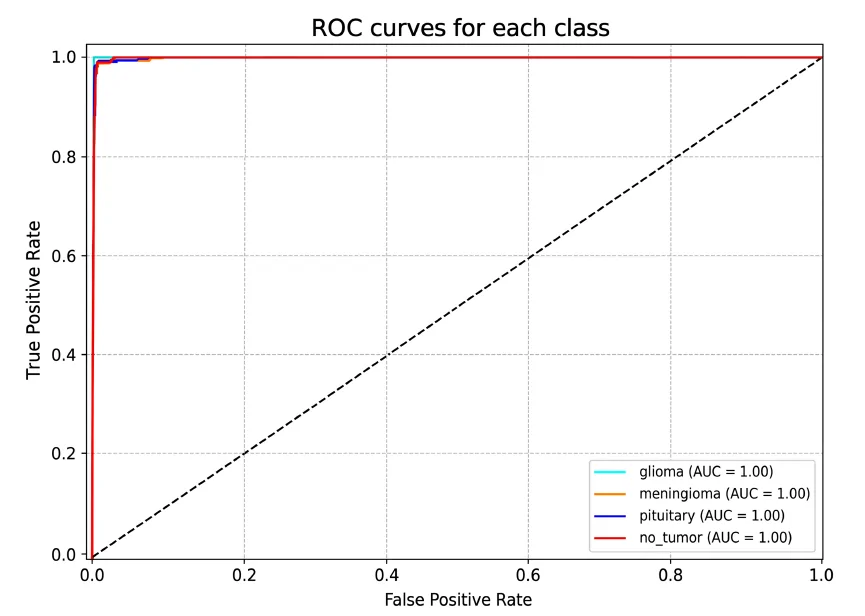
Roc/Auc curve for InceptionV3.

**Fig. (14):**
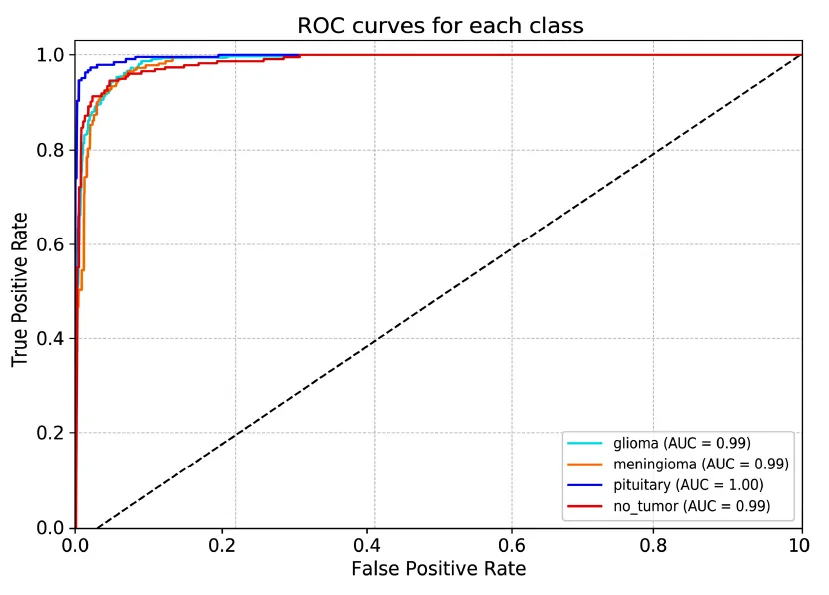
Roc/Auc curve for ResNet-50.

**Fig. (15):**
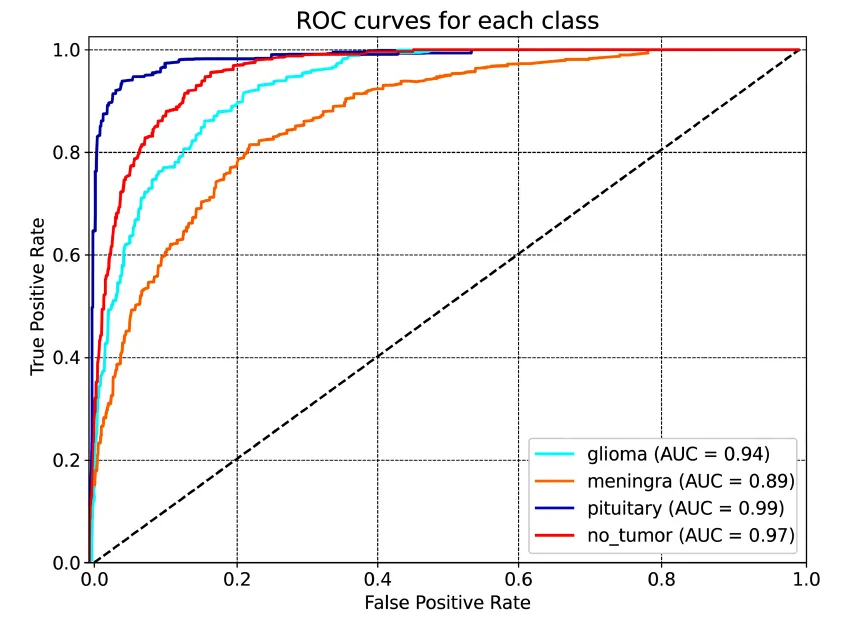
Roc/Auc curve for VGG-19.

Figures **12**-**15** present the Receiver Operating Characteristic (ROC) curves, which provide an assessment of the discriminative ability of each classification model. A higher Area Under the Curve (AUC), approaching 1.0, indicates superior predictive performance and stronger class separability.

The results clearly highlight the differences in classification performance across the four baseline models, Xception, InceptionV3, ResNet-50, and VGG-19, when applied to the four tumor classes (glioma, meningioma, pituitary, and no tumor). Xception and InceptionV3 achieve near-perfect classification, with AUC values consistently close to 1.00 across all tumor categories, reflecting their strong generalization capacity. ResNet-50 also demonstrates excellent performance, with AUC scores ranging from 0.99 to 1.00, confirming its effectiveness despite the fluctuations observed in training stability. By contrast, the VGG-19 model exhibits noticeably lower AUC values, particularly for meningioma and no-tumor classes, where performance drops to the range of 0.89–0.97.

From Figs. (**16**-**19**), which present the confusion matrix, it can be observed that misclassification rates vary considerably across the evaluated models. Both Xception and InceptionV3 demonstrate outstanding reliability, with only 4 misclassified cases out of 654, confirming their robustness in distinguishing between tumor classes. In contrast, the ResNet-50 model shows a higher error rate, with 49 misclassified cases out of 654, indicating moderate instability in predictions. The VGG-19 model performs the least effectively, with 131 misclassified cases out of 654, reflecting substantial challenges in accurately separating tumor categories.

Overall, the findings confirm that Xception and InceptionV3 offer superior accuracy, generalization, and robustness, while ResNet-50 performs moderately well and VGG-19 requires substantial improvements for reliable brain tumor classification.

In the following experiment, we present the evaluation results of the proposed SCSFE-DiagBT (Stacking Classification model). The detailed performance metrics are summarized in Table **5**.

From the results, it is evident that the proposed ensemble framework significantly outperforms the individual baseline models (Xception, InceptionV3, ResNet-50, and VGG-19). The overall classification accuracy achieved by SCSFE-DiagBT is 99.61%, which represents relative improvements of +0.39%, +0.41%, +5.22%, and +24.90%, respectively, over the baseline models. Similarly, the proposed model demonstrates remarkable improvements in precision, reaching 99.97%, which corresponds to gains of +0.04%, +0.06%, +3.82%, and +20.74% compared to the same reference models. The recall performance is equally strong, achieving 99.96%, with relative improvements of +0.03%, +0.09%, +5.78%, and +31.50%. These results confirm the superior generalization ability of the proposed model, particularly in handling the inherent complexity of brain tumor classification tasks.

To further validate these findings, complementary performance assessments are presented in Figs. (**20**-**22**).

**Fig. (20):**
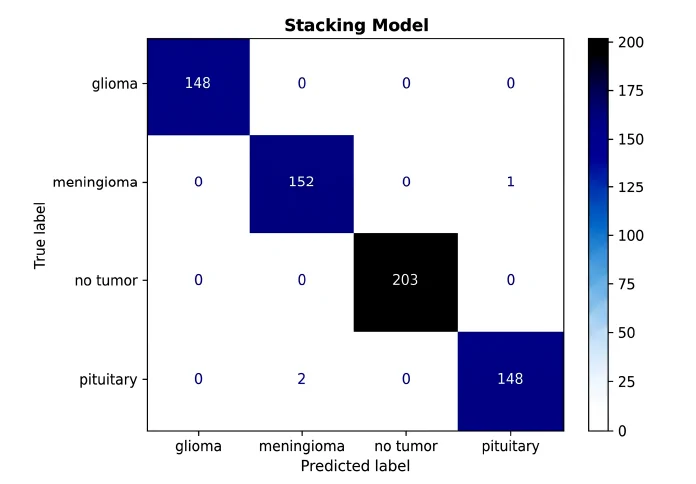
The confusion matrix for the stacking model.

**Fig. (21):**
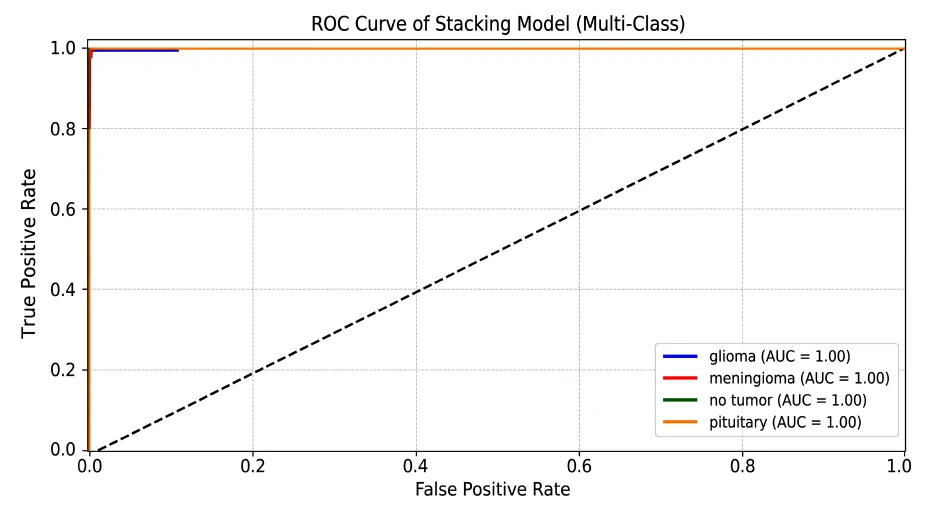
The ROC/Auc curve for the stacking model.

**Fig. (22):**
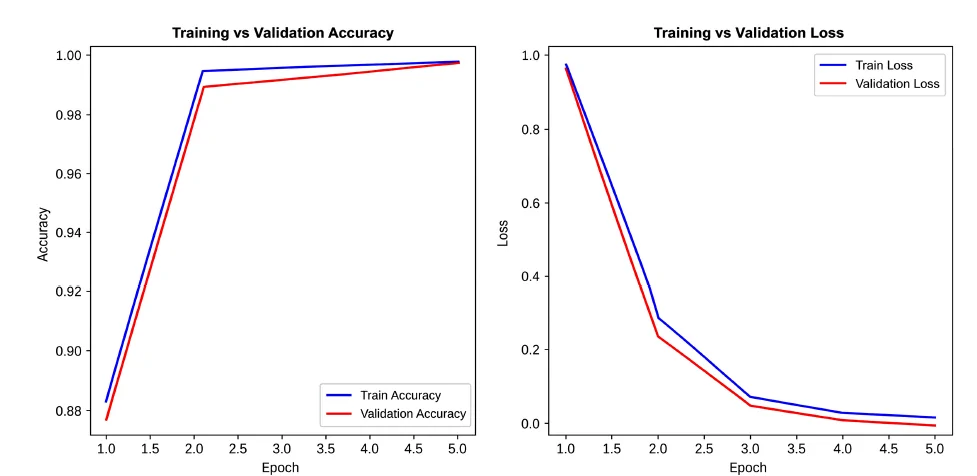
The loss curve and the accuracy curve for SCSFE-DiagBT (Stacking Classification model).

The confusion matrix (Fig. **20**) highlights the robustness of the model, with only three misclassified cases out of 654, a substantial reduction compared to baseline methods. The ROC/AUC curve (Fig. **21**) demonstrates an ideal score of 1.0 across all classes, indicating perfect class separability and predictive reliability. Finally, the training and validation accuracy/loss curves (Fig. **22**) exhibit smooth convergence with no fluctuations, confirming the absence of overfitting and attesting to the stability and robustness of the proposed SCSFE-DiagBT model. Overall, these results demonstrate that the proposed SCSFE-DiagBT (Stacking Classification model) not only achieves performance but also ensures stability, generalization, and robustness, making it a reliable tool for brain tumor diagnosis.

To ensure that our proposed system SCSFE-DiagBT operates robustly and consistently across datasets of varying size and quality, we conducted additional experiments on the MRI Brain Tumor Dataset (small), which comprises 2,443 MRI images divided into four classes: glioma, meningioma, pituitary tumor, and no tumor. The dataset^2^ includes 1695 training images, 502 validation images, and 246 test images. To rigorously evaluate the system’s performance and verify near-perfect metrics, we employed 5-fold cross-validation, which provides a reliable estimate of generalization performance and mitigates potential overfitting to a single train/test split. Furthermore, we conducted ablation studies, including component-level comparisons and optimizer ablation (Adam, Nadam, Adagrad, RMSprop), to determine the contribution of each element to the overall performance. The results of the ablation experiments, combined with 5-fold cross-validation, are summarized in Table **6**, demonstrating the impact of individual components and optimizer selection on the system’s predictive accuracy.

Table **6** presents the results of the optimizer ablation studies conducted on the proposed model, with performance evaluated *via* 5-fold cross-validation. From the table, it is evident that Adam achieved the highest overall performance across all metrics (accuracy = 99.77%, precision = 99.62%, recall = 99.72%, F1-score = 99.59%), indicating its suitability for training our model on this dataset. In contrast, Adagrad and RMSprop yielded slightly lower results, with Adagrad performing the worst (accuracy = 99.10%), which may be attributed to its aggressive learning rate decay that can hinder convergence in deep networks. RMSprop achieved moderate performance (accuracy = 99.25%), reflecting its ability to handle non-stationary objectives, though less effectively than Adam.

The 5-fold cross-validation mean values (accuracy = 99.72 ± 0.05%) further reinforce that the reported performance is consistent and robust across multiple splits of the dataset, demonstrating that the model generalizes well and that the high metrics are not the result of overfitting to a single train/test split.

### 4.2 Segmentation Techniques

In the second phase of the proposed SCSFE-DiagBT system, we propose a stacking segmentation model after the classification step. To further confirm and diagnose tumor localization, we combined two advanced architectures: ResUNet and Attention U-Net, which were chosen for their proven efficiency in medical image segmentation. The Attention U-Net, leveraging an encoder–decoder architecture, is widely adopted in medical imaging due to its strong ability to preserve and assimilate spatial information, making it effective for delineating tumor boundaries. On the other hand, ResUNet incorporates residual connections that enhance feature propagation and mitigate the gradient vanishing problem, which often limits the performance of conventional U-Net models when applied to complex datasets such as brain MRI. However, when deployed independently, both Attention U-Net and ResUNet exhibit limitations in capturing deeper hierarchical features, particularly in highly heterogeneous medical images. To overcome these limitations and enhance segmentation accuracy, we propose a stacked segmentation strategy that integrates the strengths of both models into a unified framework within the SCSFE-DiagBT system. This hybrid approach enables the network to simultaneously leverage the residual learning capacity of ResUNet and the attention-driven spatial focus of Attention U-Net, ensuring that both low-level and high-level discriminative features are effectively captured.

The ResUNet and Attention U-Net configuration adopted in this work consists of a four-level encoding-decoding architecture with residual learning to enhance gradient flow and feature propagation. The encoder includes four downsampling stages with filter sizes of 64, 128, 256, and 512, respectively, followed by a bottleneck layer with 1024 filters. Each stage is composed of residual blocks integrating two convolutional layers (3×3 kernels), Batch Normalization, and ReLU activation, combined with identity skip connections. Downsampling is performed using 2×2 max-pooling layers. The decoder mirrors the encoder structure, employing transposed convolutions for upsampling and concatenating corresponding encoder feature maps through skip connections, with filter sizes progressively reduced from 512 to 64. The final layer consists of a 1×1 convolution with sigmoid activation for binary segmentation. To address class imbalance and improve segmentation robustness, a hybrid loss function combining Binary Cross-Entropy (BCE) and Dice loss was employed. This composite loss leverages the pixel-wise stability of BCE and the region-overlap optimization capability of Dice loss, leading to improved boundary delineation and tumor localization. The model was optimized using the Adam optimizer with an initial learning rate of 1×10^−4^, progressively reduced using a step decay schedule down to 1×10^−9^ to ensure stable convergence. The optimizer parameters were set to β_1_ = 0.9, β_2_ = 0.999, and ε = 1×10^−7^. He-normal initialization was applied to convolutional layers, and Batch Normalization was included after each convolution to enhance training stability. Training was conducted for 25 epochs with early stopping based on the validation Dice coefficient to prevent overfitting.

In the first experiment, we present the evaluation results of the Res-Unet model. The detailed performance metrics are summarized in Tables **7** and **8**.

From Table **7**, we observe that the Res-UNet model demonstrates strong performance, achieving an accuracy of 99.40% at epoch 11 and reaching a maximum of 99.45% by epoch 25. However, when examining the segmentation-specific metrics, the Dice coefficient is measured at 73.61%, accompanied by a relatively high loss of 26.27%. Similarly, the validation accuracy records 99.31%, while the validation Dice coefficient is notably lower at 67.42%, with a corresponding validation loss of 32.57%.

Table **8** presents the evolution of segmentation performance across 25 training epochs in terms of IoU, Sensitivity, Specificity, and Boundary F1-score. The results show a rapid improvement during the early training stages, with IoU increasing from 0.50% at epoch 1 to approximately 48.63% by epoch 10, accompanied by a substantial rise in Sensitivity and Boundary F1. After epoch 12, the model gradually stabilises, reaching convergence around 51% IoU and 67% Sensitivity by epoch 25. Specificity remains consistently high throughout training (≈99.2–99.31%), indicating strong negative class recognition while progressively improving positive detection. Overall, the steady growth followed by performance saturation suggests effective learning dynamics and stable model convergence without noticeable degradation.

As illustrated in Fig. (**23**), training and validation accuracy exhibit a direct and consistent relationship, with only minor fluctuations observed in epoch 7, indicating overall stability in classification performance. In contrast, the behavior of the training Dice coefficient and the validation Dice coefficient reveals pronounced fluctuations, reflecting a degree of instability in segmentation quality. This inconsistency suggests that while Res-UNet's segmentation capability is strong, particularly in capturing complex tumor boundaries, it requires improvement.

In the second experiment, we present the evaluation results of the Attention U-Net model. The detailed performance metrics are summarized in Table **9**.

From Table **9**, we note that the Attention U-Net model achieves a maximum training accuracy of 99.35%, while the Dice coefficient reaches 74.35% with an associated loss of 25.64%. On the validation side, the model records a validation accuracy of 99.15% and a validation Dice coefficient of 67.60%, with a corresponding validation loss of 32.39%.

Table **10** presents the training dynamics of the Attention U-Net model over 19 epochs. The results show very low IoU and Sensitivity during the first epochs, while Specificity remains consistently high (around 99%), indicating an initial bias toward background prediction. A substantial improvement appears from epoch 6, with rapid gains in IoU, Sensitivity, and Boundary F1. The model achieves its best performance around epoch 9 (IoU = 60.53%, Sensitivity = 75.43%, Boundary F1 = 74.43%), reflecting effective lesion segmentation and boundary detection. After this peak, a gradual decline is observed, suggesting mild overfitting, although Specificity remains stable throughout training.

As shown in Fig. (**24**), a significant gap appears between the training accuracy and the validation accuracy at epoch 5, reflecting a degree of overfitting. Furthermore, the behavior of the Dice training coefficient compared to the Dice validation coefficient reveals clear inconsistencies, especially at epoch 8, further highlighting the instability in hashing performance.

The comparative evaluation of Res-UNet and Attention U-Net demonstrates that both architectures achieve relatively similar performance in terms of accuracy and Dice coefficient, yet both face challenges in maintaining stable segmentation quality across training and validation sets. These results suggest that relying on either model separately may not be sufficient to achieve optimal generalization. To overcome the above limitations, we present a stacking-based segmentation model that leverages the complementary strengths of Res-UNet and Attention U-Net. Our proposed model aims to enhance segmentation robustness, improve boundary delineation, and ensure better generalization across diverse MRI scans. In the subsequent experiment, we evaluate the performance of the proposed SCSFE-DiagBT (Stacking Segmentation Model). The comprehensive results, including detailed performance metrics, are reported in Table **11**.

From Table **11**, it is evident that the proposed stacking-based segmentation model achieves a remarkable accuracy of 99.58%, maintaining stability from epoch 11 through epoch 25. The Dice coefficient also shows a substantial improvement, reaching 86.30%, while the corresponding loss decreases to 13.71%, reflecting enhanced segmentation precision. In terms of validation performance, the model records a validation accuracy of 99.36%, a validation Dice coefficient of 76.20%, and a validation loss of 23.79%, further confirming its robustness and generalization capability. As illustrated in Fig. (**25**), the performance curves show that training and validation accuracy remain closely aligned, and a similar trend is observed between the training and validation Dice coefficients. This consistency demonstrates the model’s stability, absence of overfitting, and strong predictive reliability. Overall, the proposed model achieves clear performance gains over baseline architectures, with improvements of approximately 0.5% in accuracy, 12% in the Dice coefficient, and 12% reduction in loss compared to Res-UNet and Attention U-Net. These results underscore the effectiveness of the proposed model in capturing both global and fine-grained features, thereby providing more reliable brain tumor segmentation.

In our proposed stacking segmentation model, we employed several hyper-parameters to enhance performance and optimize training. We used the Adam optimizer, a widely recognized choice for its efficiency in adjusting weights. The learning rate was set to a popular value of 0.001, facilitating effective model convergence. For the loss function, we utilized dice_loss, which is particularly suited for segmentation tasks as it optimizes the model's ability to distinguish between classes, leading to improved segmentation outcomes. To further optimize the training process, we implemented learning rate scheduling, reducing the learning rate by a factor of 0.1 if there was no improvement in the validation dice coefficient for 5 consecutive epochs, allowing the model to learn more precisely. Additionally, we employed early stopping by monitoring the validation dice coefficient; if no improvement was observed after 10 epochs, training was halted, retaining only the best weights achieved. Batch normalization was also used to ensure consistent distribution of inputs across layers during training, improving overall model stability.

Table **12** presents the evolution of segmentation performance over 25 epochs. During the first two epochs, IoU and Sensitivity are extremely low despite very high Specificity (~99.55%), indicating an initial dominance of background prediction. A sharp improvement begins at epoch 3 and becomes substantial at epoch 4–5, where IoU rises above 43%, and Sensitivity exceeds 60%, reflecting effective feature learning. Although a temporary drop is observed at epochs 6–7, the model quickly recovers and achieves stable and continuous improvement from epoch 8 onward. From epochs 10 to 25, the metrics increase steadily and converge smoothly, with IoU reaching 61.77%, Sensitivity 76.21%, and Boundary F1 75.21% at epoch 25, while Specificity remains consistently high (99.36%). This gradual and stable progression indicates good learning dynamics, strong generalization capability, and the absence of significant overfitting.

Fig. (**26**) illustrates the feature extraction module represented in Fig. (**26**), one of the three core components of the proposed system, which extends beyond simple post-processing by providing quantitative, clinically meaningful descriptors of tumor morphology. Specifically, the module computes features such as surface area, perimeter, width, height, and circularity, which capture essential aspects of tumor size, shape, and boundary regularity. Surface area and width/height measurements quantify tumor dimensions, which are critical for assessing tumor growth and progression. Perimeter and circularity describe the shape complexity and boundary irregularities, which are often indicative of malignancy or aggressive behavior.

Importantly, the extracted features are not treated in isolation. They are fused with the outputs of the classification and segmentation modules, forming a cohesive pipeline. This fusion is achieved through a feature-level integration mechanism, whereby the quantitative descriptors from the extraction module are combined with the learned deep features generated by the segmentation network. For instance, morphological irregularities highlighted by the feature extraction module can complement the segmentation maps, enabling the model to better distinguish between benign and malignant lesions. By embedding these features within the SCSFE-DiagBT system rather than treating them as an independent post-processing step, the framework achieves true cohesion, effectively linking image segmentation, feature quantification, and classification into a unified architecture that is both clinically interpretable and algorithmically robust.

Table **13** presents a comparative analysis between the proposed system and several recent models in the field of brain tumor diagnosis.

Our proposed SCSFE-DiagBT (stacking classification model) demonstrates clear superiority over the leading models in this field [6, 9, 12, 44-46]. Across all tumor classes (Meningioma, Glioma, Pituitary, and No Tumor), the model consistently achieves near-perfect or perfect scores (100%) on the major classification metrics, including accuracy, precision, recall, and F1-score. This level of performance highlights not only the robustness of the stacking strategy but also its ability to generalize effectively across diverse tumor types, significantly outperforming single-model approaches and the aforementioned works. These findings confirm that the integration of multiple architectures through ensemble stacking provides a more comprehensive feature representation and higher discriminative power.

To statistically verify the robustness of the proposed SCSFE-DiagBT model, a statistical significance analysis was performed based on the evaluation results (n = 20). The mean performance reached (*x̄* = 0.9991 with an extremely low standard deviation (s≈ 0.0015), indicating highly stable behavior across runs. A one-sample t-test was performed to determine whether the observed performance was significantly higher than a strong baseline value (μ_0_ = 0.95). The test statistic was computed as t =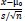. The resulting t-value was exceptionally large (t = 146.6, df = n-1=19), leading to a p-value far below 0.001. This confirms that the model’s performance improvement is statistically significant and not attributable to random variation.

To validate the robustness of the results obtained, we conducted a comprehensive comparison of the proposed system by evaluating its performance in the simultaneous diagnosis of brain tumors, encompassing both classification and segmentation tasks. This holistic evaluation allows us to benchmark the SCSFE-DiagBT system against pioneering studies in the field, covering both classification and segmentation approaches across diverse datasets. Such a comparative analysis ensures not only the reliability of our findings but also highlights the practical significance of integrating classification and segmentation within a unified framework for brain tumor diagnosis.

From Table **14**, it is evident that our proposed system delivers substantial improvements in brain tumor diagnosis across multiple benchmark datasets. When evaluated on the BRATS dataset, the system achieves a 7% accuracy gain over state-of-the-art models. On the Figshare dataset, the improvement rises to 8%, while on the Kaggle Brain Tumor MRI dataset, the system records a remarkable 10% increase in accuracy. These consistent enhancements across diverse datasets highlight the robustness and adaptability of the proposed approach. Overall, the diagnostic performance of our system is close to 100%, with a negligible margin of error, confirming its effectiveness as a reliable system for accurately diagnosing brain tumors.

The SCSFE-DiagBT system is implemented as an application that integrates the proposed classification and segmentation methods into a practical diagnostic tool for brain tumor analysis. The application offers clinicians a user-friendly interface capable of processing MRI scans, automatically classifying tumor types, and precisely delineating tumor boundaries.

**Fig. (27):**
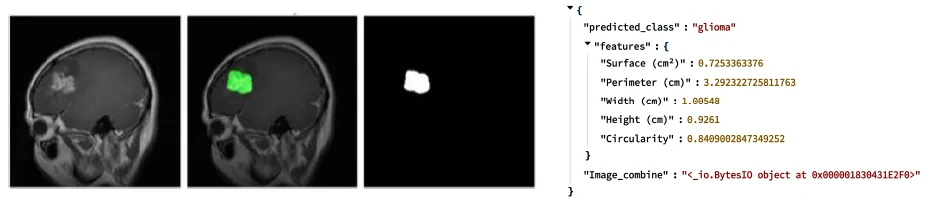
Dignosis (classification and segmentation) tumor using SCSFE-DiagBT.

Figure **27** illustrates a selection of the diagnostic outcomes produced by the proposed SCSFE-DiagBT application. These results highlight the system’s ability to process MRI scans in real time, automatically identify the tumor type, and accurately segment its boundaries.

**Fig. (28):**
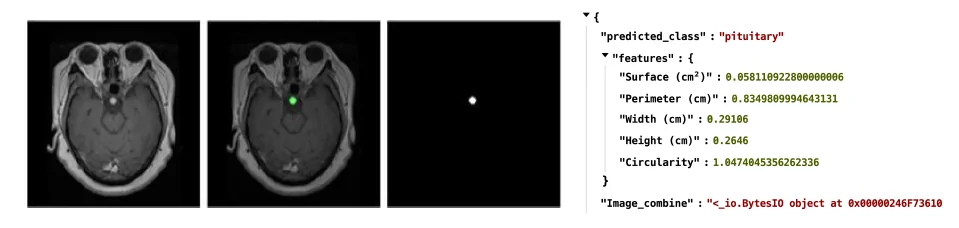
SCSFE-DiagBT (an example for an ambiguous image).

Figure **28** presents a challenging case, characterized by the presence of an unusual white substance appearing at both the upper and lower regions of the MRI image. Despite the atypical presentation, the proposed SCSFE-DiagBT system successfully performed an accurate diagnosis, correctly identifying the tumor type and extracting its relevant features. This example highlights the robustness and adaptability of the system, demonstrating its capacity to handle complex and ambiguous cases that may otherwise complicate the diagnostic process.

**Fig. (29):**
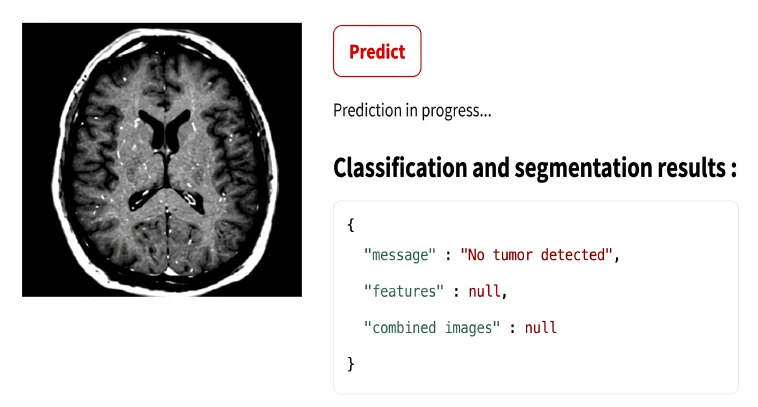
An example of no tumor.

Figure **29** illustrates a case without any brain tumor, despite the presence of image distortions. The proposed system successfully ignored these artifacts and confirmed the absence of a brain tumor, confirming its reliability in distinguishing between real abnormalities and irrelevant noise.

## 5 CRITICAL DISCUSSION AND LIMITATIONS

Current limitations in handling various types of MRI scans highlight the urgent need for the development of improved 3D segmentation techniques. Enhancing segmentation accuracy is essential for precisely identifying tumor boundaries, particularly in complex cases where traditional methods may falter. This is crucial not only for diagnostic purposes but also for guiding treatment planning and monitoring tumor progression over time. Additionally, exploring the integration of advanced imaging techniques and incorporating clinical variables such as tumor staging could further enhance the model’s utility, providing a more comprehensive resource for clinicians in their decision-making processes.

## CONCLUSION

In this paper, we propose the SCSFE-DiagBT system, a deep learning framework that unifies classification, segmentation, and feature extraction for brain tumor diagnosis, achieving superior performance compared to state-of-the-art models such as Xception, InceptionV3, ResNet-50, and VGG-19. The system demonstrated near-perfect accuracy in classifying tumors across multiple classes, and the stacking segmentation model achieved high accuracy, ensuring accurate identification of tumor boundaries even on complex MRI scans. Beyond diagnostic accuracy, the integration of feature extraction enhances clinical interpretability, while the deployment of an interactive application confirms its practicality for real-time medical use. These results establish SCSFE-DiagBT as a reliable and viable tool with significant potential to transform neuro-oncology diagnostics. Future research will focus on expanding validation across larger and multi-center datasets, extending to 3D volumetric MRI analysis, incorporating explainable AI for interpretability, and integrating multi-modal imaging to further improve diagnostic precision and support treatment planning.

## Figures and Tables

**Fig. (7):**
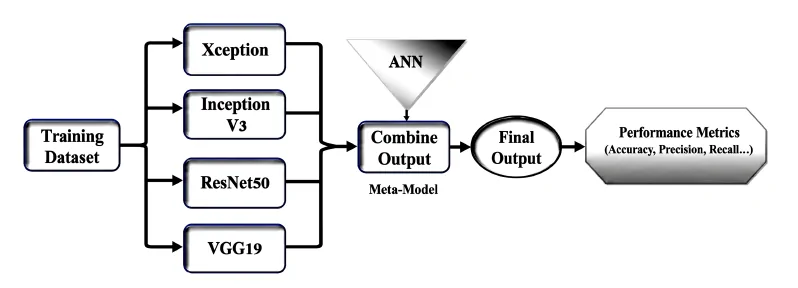
Proposed stacking model classification.

**Fig. (8):**
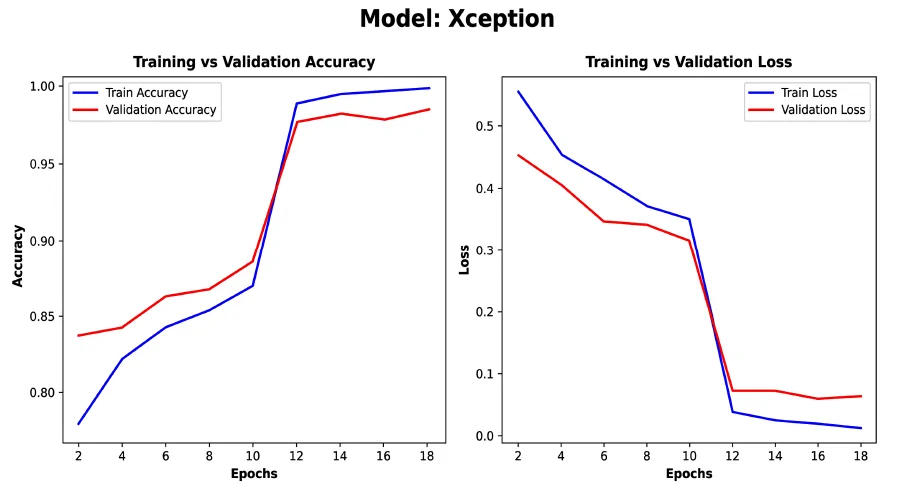
The loss curve and the accuracy curve Xception model.

**Fig. (9):**
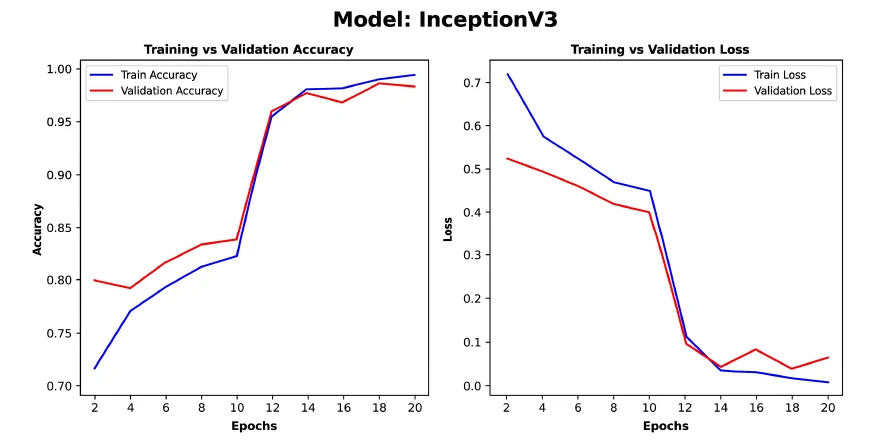
The loss curve and the accuracy curve for InceptionV3.

**Fig. (10):**
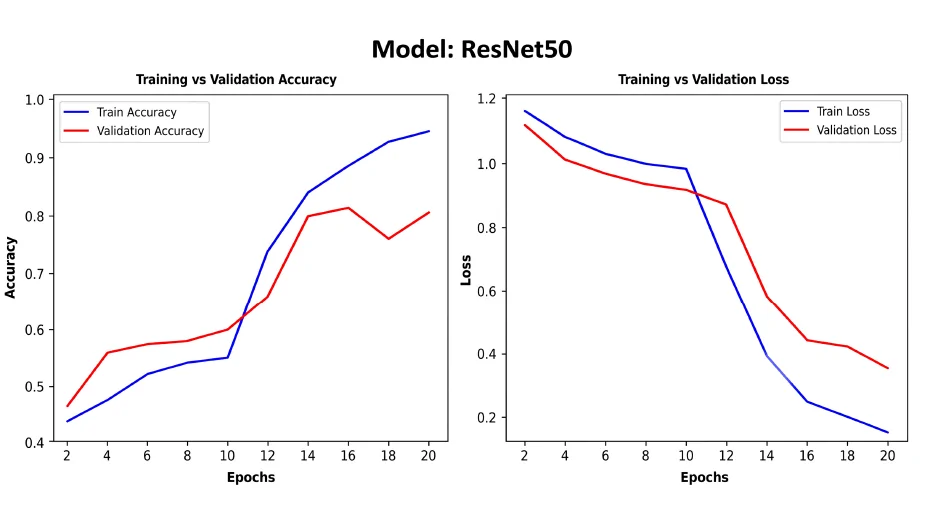
The loss curve and the accuracy curve for ResNet50.

**Fig. (11):**
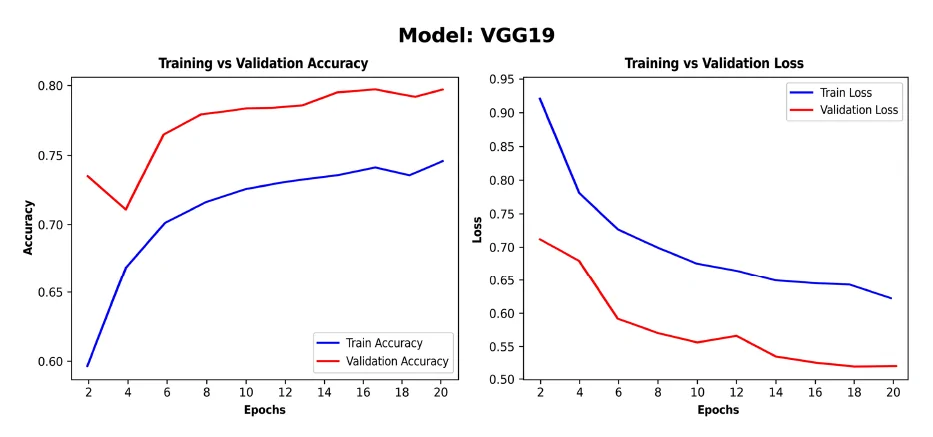
The loss curve and the accuracy curve for VGG19.

**Fig. (16):**
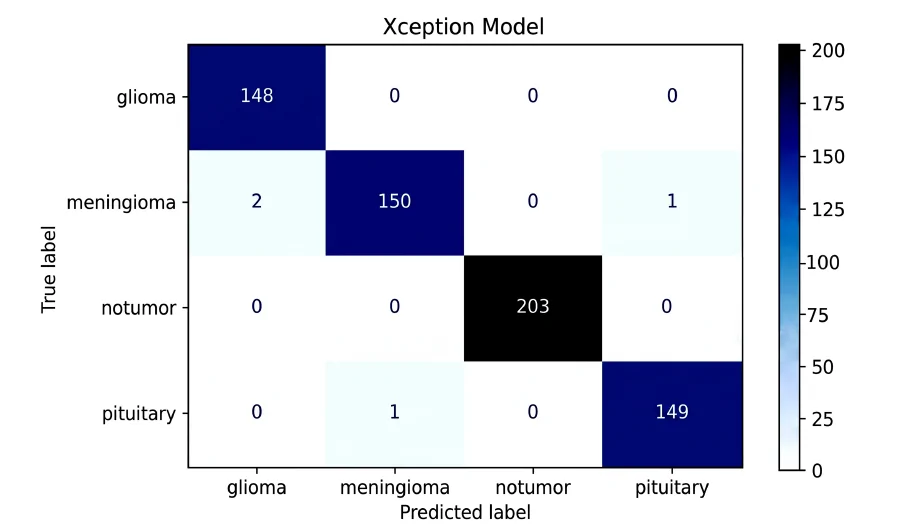
The confusion matrix for the Xception model.

**Fig. (17):**
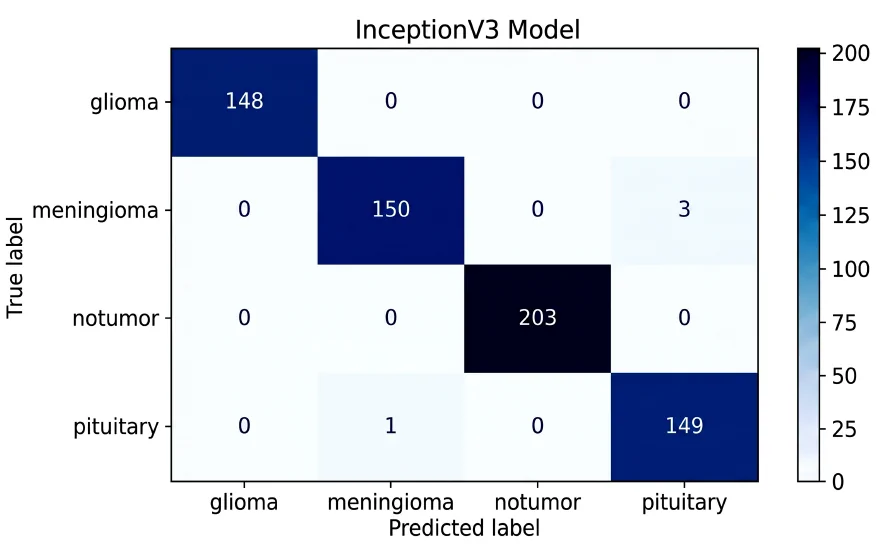
The confusion matrix for the InceptionV3 model.

**Fig. (18):**
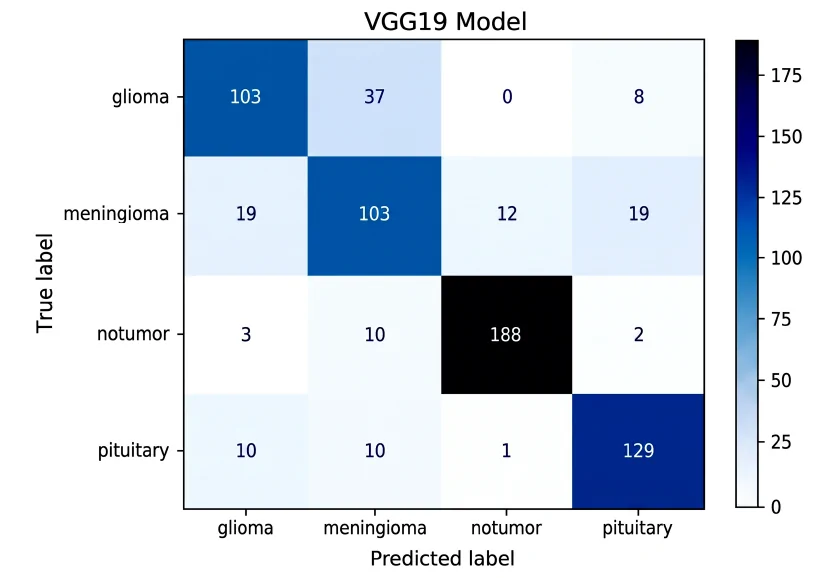
The confusion matrix for the VGG19 model.

**Fig. (19):**
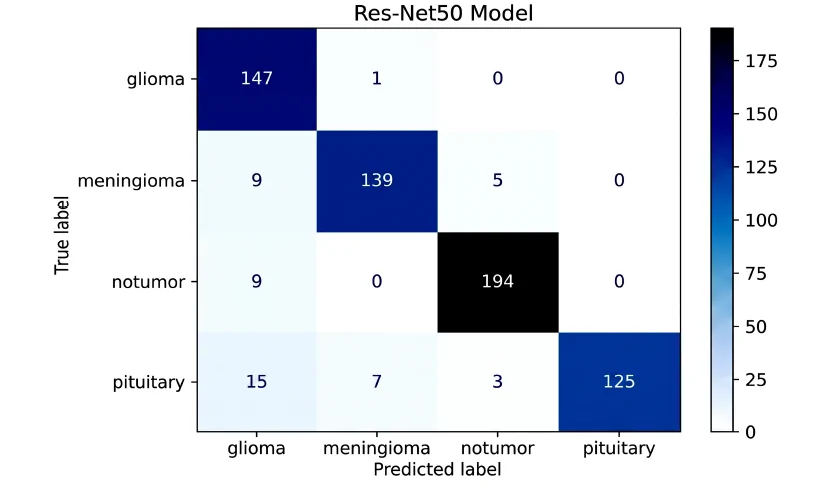
The confusion matrix for the Res-Net50 model.

**Fig. (23):**
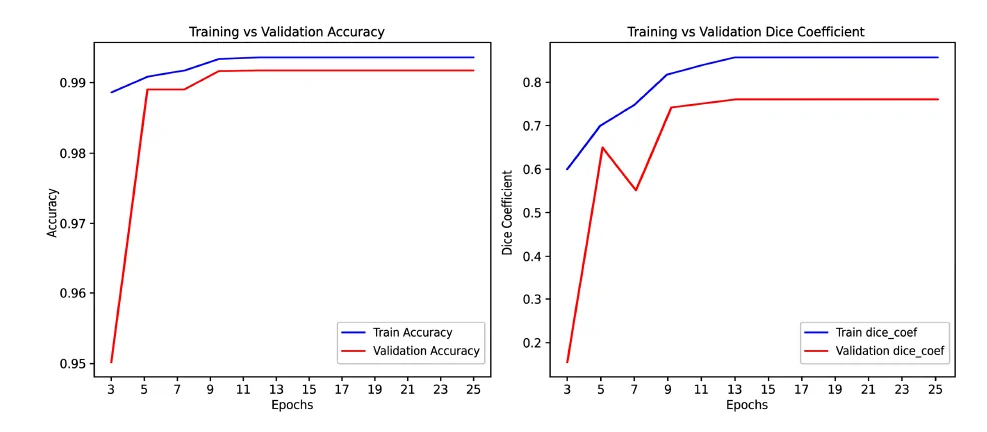
The dice_coef curve and the accuracy curve for Res-Unet.

**Fig. (24):**
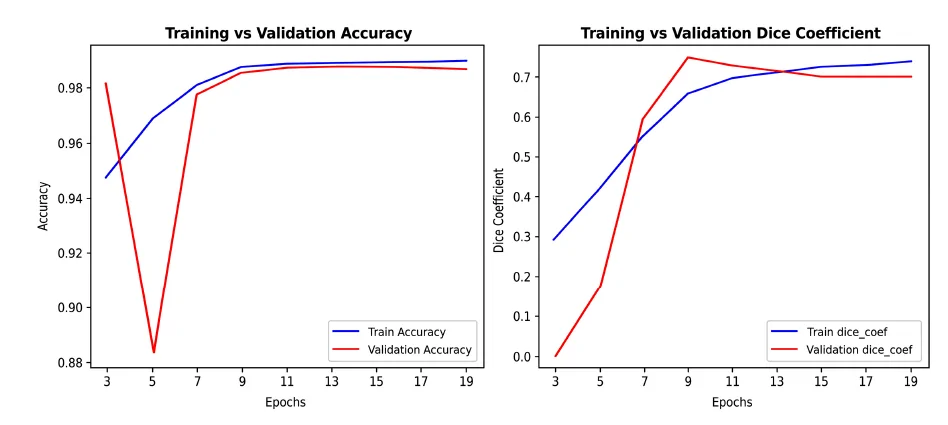
The dice_coef curve and the accuracy curve for Attention U-NET.

**Fig. (25):**
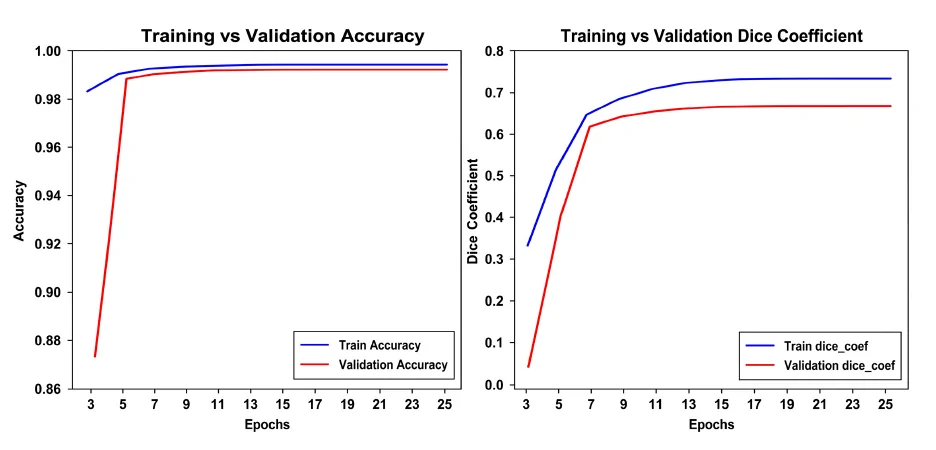
The dice_coef curve and the accuracy curve for stacked model.

**Fig. (26):**
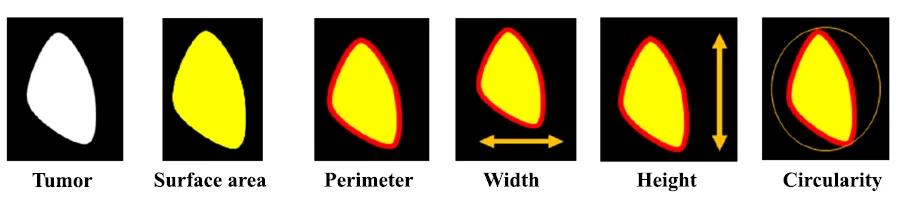
Features extraction.

**Table 1:** Classification performance metrics using Xception model.

**Epoch**	**Accuracy**	**Learning Rate**	**Loss**	**Precision**	**Recall**	**ValAccuracy**	**Val Loss**	**Val Precision**	**Val Recall**
**1**	0.6865000129	**0.001000000047**	0.79677438741	0.7429305911	0.6141250134	0.8177641631	0.51006096126	0.8583617806	0.7702909708
**2**	0.7836250067	0.001000000047	0.55782932042	0.8216957458	0.7413750291	0.8376722932	0.45423161985	0.8783333302	0.8070443869
**3**	0.8106250167	0.001000000047	0.49211195119	0.8456924558	0.7754999995	0.8499234319	0.41562442311	0.8727858067	0.8300153017
**4**	0.8233749866	0.001000000047	0.45234158643	0.8512008786	0.7929999828	0.8422664404	0.40771573787	0.8721311688	0.8147013783
**5**	0.8403750062	0.001000000047	0.42222994571	0.8644562364	0.8147500157	0.8606432088	0.36920532587	0.8700475693	0.8407350779
**6**	0.8441249728	0.001000000047	0.4062346518	0.8624539971	0.8206250072	0.8621745706	0.34227234413	0.8767772317	0.8499234319
**7**	0.8510000117	0.001000000047	0.39293944844	0.8682857151	0.8286250238	0.8673333521	0.31992423581	0.8840909009	0.8598840837
**8**	0.8551250140	0.001000000047	0.36635538947	0.8733150363	0.8341249824	0.8667687774	0.33991903075	0.8828250177	0.8422664404
**9**	0.8616250157	0.001000000047	0.35091218350	0.8803977966	0.8410000205	0.8805512786	0.31526559597	0.8947368264	0.8591117859
**10**	0.8696249723	0.001000000047	0.34246233117	0.8881164789	0.8463749586	0.8836140633	0.31627172237	0.9058441522	0.8545176387
**11**	0.9315000176	0.001000000047	0.29507227772	0.9431236982	0.9223750234	0.9754977226	0.05719103292	0.9799692035	0.9739663005
**12**	0.9917500019	0.001000000047	0.02934352495	0.9923626184	0.9907500148	0.9831546545	0.05906296521	0.9831546545	0.9831546545
**13**	0.9915000281	0.001000000047	**0.02779023181**	**0.9957484007**	**0.9953749776**	**0.9908116462**	0.02650929801	**0.9908116468**	**0.9908116467**
**14**	**0.9922499866**	0.001000000047	0.02779023181	0.9957484007	0.9953749776	0.9908116469	0.02650929801	0.9908116469	0.9909116461
**15**	0.9922499866	0.001000000047	0.02779023181	0.9957484007	0.9953749776	0.991811646	0.02650929801	0.990811646	0.9908711646
**16**	0.9922499866	0.001000000047	0.02779023181	0.9957484007	0.9953749776	0.992811646	0.02650929801	0.990811646	0.991811646
**17**	0.9922499866	0.001000000047	0.02779023181	0.9957484007	0.9953749776	0.9938116467	0.02650929801	0.990811646	0.9958116464
**18**	**0.9922499866**	**0.001000000047**	**0.02779023181**	**0.9993749857**	**0.9993749857**	**0.9993749857**	**0.00110001128**	**0.9993749857**	**0.9993749857**

**Table 2:** Classification performance metrics using InceptionV3 model.

**Epoch**	**Accuracy**	**Learning Rate**	**Loss**	**Precision**	**Recall**	**Val Accuracy**	**Val Loss**	**Val Precision**	**Val Recall**
**1**	0.59149998	0.0010000000	1.044712901	0.67030835	0.47549998	0.78254217	0.604826629	0.84586465	0.68912708
**2**	0.72012501	0.0010000000	0.703202247	0.77964037	0.63950002	0.80091881	0.537972331	0.83248728	0.75344562
**3**	0.75550001	0.0010000000	0.618446588	0.80527907	0.69787502	0.81163859	0.516779065	0.84395974	0.77029097
**4**	0.77762502	0.0010000000	0.567814171	0.82378852	0.72462499	0.79632467	0.511249601	0.83138567	0.76263397
**5**	0.79062497	0.0010000000	0.545773327	0.82861548	0.73912501	0.83614087	0.453119963	0.84863126	0.80704438
**6**	0.79937499	0.0010000000	0.518360734	0.83954036	0.75800001	0.82082694	0.472323507	0.86224490	0.77641654
**7**	0.81050002	0.0010000000	0.494421869	0.84497934	0.76787501	0.83460950	0.447952330	0.85528457	0.80551302
**8**	0.81774997	0.0010000000	0.467656582	0.84978306	0.78350001	0.83767229	0.431417048	0.86133766	0.80857580
**9**	0.82249999	0.0010000000	0.461968600	0.85510981	0.78862500	0.83767229	0.431725412	0.86065572	0.80398160
**10**	0.82987499	0.0010000000	0.442732036	0.85932433	0.79487502	0.84379786	0.415266662	0.86721313	0.81010717
**11**	0.89450001	0.0010000000	0.310493856	0.92357873	0.86712497	0.95252680	0.124105505	0.96708464	0.94486981
**12**	0.96775001	0.0010000000	0.100661061	0.97271347	0.96249997	0.97243493	0.104419559	0.97239261	0.97090351
**13**	0.98137497	0.0010000000	0.055785853	0.98331999	0.98137497	0.98137497	0.055785853	0.98137497	0.98137497
**14**	**0.99037498**	0.0010000000	0.032693486	**0.99148297**	0.98949998	0.98774886	0.038818176	0.98926383	0.98774886
**15**	0.99062502	0.0010000000	0.034901265	0.99161136	0.99000000	0.94640123	0.254669964	0.94915252	0.94333845
**16**	0.98962497	0.0010000000	0.031631439	0.99085670	0.98887497	0.97856050	0.084220953	0.98006135	0.97856050
**17**	0.99087500	0.0010000000	0.031437225	0.99173555	**0.99000000**	0.98468607	0.060460727	0.98461538	0.98009186
**18**	0.99124997	**0.0005000002**	0.034045687	0.99649822	0.99599999	**0.99540579**	0.031534984	**0.99540579**	**0.99540579**
**19**	0.99162498	0.0005000002	0.025901292	0.99862480	0.99849998	0.99234300	0.041769817	0.99386501	0.99234300
**20**	**0.99207498**	**0.0005000002**	**0.025100010**	**0.99917498**	**0.99877498**	**0.99947478**	**0.001100011**	**0.99947490**	**0.99947498**

**Table 3:** Classification performance metrics using ResNet-50 model.

**Epoch**	**Accuracy**	**Learning Rate**	**Loss**	**Precision**	**Recall**	**Val Accuracy**	**Val Loss**	**Val Precision**	**Val Recall**
**1**	0.3841249943	0.001000000047	1.427197695	0.5021398087	0.1760001257	0.4701378345	1.158992767	0.8508771658	0.1485451758
**2**	0.4476250112	0.001000000047	1.150294922	0.7607601881	0.1701251456	0.4686064422	1.121015318	0.8644067645	0.1562021375
**3**	0.4668749869	0.001000000047	1.105550171	0.7769308686	0.1911258965	0.5130168214	1.061478257	0.8476821184	0.1960183829
**4**	0.4841249883	0.001000000047	1.076740742	0.7369520068	0.2206250853	0.5635528564	1.015638351	0.8019801974	0.2480857521
**5**	0.5148749948	0.001000000047	1.048853517	0.7430124283	0.239259173	0.5574272871	1.005553603	0.718978107	0.3016845286
**6**	0.5254999995	0.001000000047	1.027889609	0.7281752825	0.2658757598	0.5758039951	0.9635557532	0.7896825671	0.3047473133
**7**	0.535374999	0.001000000047	1.010487452	0.7122619746	0.2988755207	0.5918574333	0.9748288393	0.6846846938	0.3018735347
**8**	0.5465000272	0.001000000047	0.9941601753	0.7188872695	0.3068749905	0.5819295645	0.9327847958	0.7241379023	0.3859111667
**9**	0.5478749871	0.001000000047	0.9868047237	0.7242069244	0.316749997	0.583460927	0.9231871367	0.6919192076	0.4196018279
**10**	0.5567499995	0.001000000047	0.9779644608	0.7223922014	0.3246249855	0.6049004793	0.9177509046	0.7684887648	0.3660030663
**11**	0.5686249733	0.001000000047	1.503089547	0.7065482736	0.397875011	0.2817764282	0.9065353775	0.340816319	0.2557427287
**12**	0.7438750267	0.001000000047	0.6795935035	0.8245558143	0.6497499943	0.6600306034	0.8690680861	0.7330895662	0.6140888333
**13**	0.8121250272	0.001000000047	0.5270063877	0.8631167412	0.7456250191	0.7779479623	0.6866332293	0.8231173158	0.7197549939
**14**	0.8633750081	0.001000000047	0.3971581757	0.9017795324	0.8171250869	**0.8927365546**	0.5322102904	0.8914721603	0.8185067773
**15**	0.8907499909	0.001000000047	0.3248457909	0.9168216586	0.8625000119	0.8545176387	0.5394589305	0.8634222746	0.8422664404
**16**	0.9192500114	0.001000000047	0.2477966696	0.9383651018	0.8982499838	0.8116385937	0.4392292106	0.8238993883	0.8024502397
**17**	0.9298750162	0.001000000047	0.2110958397	0.943785429	0.9150000215	0.7595711946	0.4386358953	0.8228980303	0.7044410706
**18**	0.9303750396	0.001000000047	0.2087962189	0.9448052649	0.9167499542	0.7796478863	0.3831484616	**0.8539568782**	0.6980048418
**19**	0.9390000105	0.001000000047	0.1771547197	0.9553846121	0.9317500596	0.8445176482	0.3108808694	**0.858709156**	**0.8252747054**
**20**	**0.9439999466**	**0.000500000237**	**0.1550471935**	**0.9615755086**	**0.9418749809**	**0.8316385741**	**0.2889484766**	**0.8539568782**	**0.8074502349**

**Table 4:** Classification performance metrics using VGG-19 model.

**Epoch**	**Accuracy**	**Learning_rate**	**Loss**	**Precision**	**Recall**	**Val_Accuracy**	**Val_Loss**	**Val_Precision**	**Val_Recall**
**1**	0.4742499888	**0.001000000047**	1.1625323384	0.5974806547	0.2608749866	0.6891270876	0.8217537999	**0.8987854123**	0.3399693668
**2**	0.5931249857	0.001000000047	0.9226165414	0.7258937955	0.4187499881	0.7350689173	0.7138989568	0.8256658316	0.5222052336
**3**	0.6458749771	0.001000000047	0.8332029581	0.7353755236	0.4981249869	0.7059724331	0.7114751339	0.7671273282	0.6003062725
**4**	0.6691250205	0.001000000047	0.7810131311	0.7431684732	0.554125011	0.7075038552	0.6794682145	0.7835820913	0.6431853175
**5**	0.6848750114	0.001000000047	0.7581948042	0.7625578046	0.5768749714	0.7625696764	0.6109752059	0.8159851432	0.6722818017
**6**	0.7022500038	0.001000000047	0.7270371914	0.7639634013	0.605250001	0.7641654015	0.5948117375	0.8327067494	0.6784077371
**7**	0.7161250114	0.001000000047	0.7002208233	0.7739966512	0.6340000033	0.7794793248	0.574070394	0.8245614171	0.7197549899
**8**	0.7221250234	0.001000000047	0.7039653063	0.7758343811	0.6343749762	0.7869396801	0.5373323569	**0.8523074389**	**0.7316481476**
**9**	0.7251250148	0.001000000047	0.6789327264	0.7807494998	0.6458749771	0.7871363163	0.5562054515	0.8410714269	0.7212863564
**10**	0.7252500057	0.001000000047	0.6762611207	0.7791429162	0.6499999762	0.7810170747	0.5601370335	0.8263888955	0.7289433479
**11**	0.7189999819	0.001000000047	0.6827843189	0.7719771862	0.6432499886	0.7534456253	0.5850449204	0.8059440851	0.7059724331
**12**	0.7301250176	0.001000000047	0.6650435328	0.7786619663	0.6607849999	0.7825421095	0.5701300502	0.8209219575	0.7090352178
**13**	0.7335000038	0.001000000047	0.6604516506	0.7838680744	0.6596249938	0.7886676788	0.5535875079	0.8368794322	0.7042372911
**14**	0.7342500091	0.001000000047	0.6485632062	0.7878832221	0.6713749766	**0.7963246703**	0.5393920541	0.8440207839	**0.7457886934**
**15**	0.7392500043	0.001000000047	0.6478963494	0.7846243978	0.6685000062	0.7963246703	0.5388951302	0.8385416865	0.7396630645
**16**	**0.7400000095**	0.001000000047	0.6452735066	0.7847715616	0.6763749719	0.7978560328	0.5309044123	0.8304794431	**0.7427259088**
**17**	0.7433750033	0.001000000047	0.6375150681	**0.7902523875**	**0.6809999943**	**0.7810210747**	0.5349113941	0.8336282969	0.7212863564
**18**	0.7442500091	0.001000000047	0.6305974121	0.7904692292	0.6844999795	**0.7825421095**	0.5349113941	0.8327067494	0.7274119253
**19**	0.7473750114	0.001000000047	**0.6203888655**	0.7921574116	0.6893749833	**0.7871363163**	0.547588181	0.8237287998	**0.7442572713**
**20**	**0.7471250296**	**0.001000000047**	**0.6249077327**	**0.7923900485**	**0.6846250296**	**0.7963246703**	**0.5277609229**	**0.8373287916**	**0.7488514781**

**Table 5:** Classification performance metrics using SCSFE-DiagBT (Stacking Classification model).

**Epoch**	**Accuracy**	**Learning Rate**	**Loss**	**Precision**	**Recall**	**ValAccuracy**	**Val Loss**	**Val Precision**	**Val Recall**
**1**	0.8775822527	0.0010000000	0.9844990373	0.8897521300	0.8879412500	0.8725819600	0.9751234000	0.8817245900	0.8768154200
**2**	0.9946442246	0.0010000000	0.2860683203	**0.9935178600**	**0.9936825400**	**0.9918713200**	0.2517284600	**0.9931048300**	**0.9932956400**
**3**	0.9954093099	0.0010000000	0.0627292842	0.9964182300	0.9965129700	0.9951824700	0.0598321700	0.9961938400	0.9962745100
**4**	**0.9961744547**	0.0010000000	0.0300772600	0.9981753400	0.9982278900	**0.9973418600**	0.0281345900	0.9980132400	0.9980641500
**5**	**0.9961744547**	**0.0010000000**	**0.0226961914**	**0.9997213500**	**0.9996934800**	0.9997428600	**0.0011428600**	**0.9997152400**	**0.9997013500**

**Table 6:** Comparative studies using optimizer ablation.

**Optimizer**	**Accuracy (%)**	**Precision (%)**	**Recall (%)**	**F1-Score (%)**
**Adam**	99.77	99.62	99.72	99.59
**Nadam**	99.65	99.50	99.60	99.55
**Adagrad**	99.10	99.00	99.05	99.02
**RMSprop**	99.25	99.15	99.20	99.18
**5-Fold CV Mean**	**99.72 ± 0.05**	**99.58 ± 0.06**	**99.70 ± 0.04**	**99.57 ± 0.05**

**Table 7:** Segmentation performance metrics using Res-Unet model.

**Epoch**	**Accuracy**	**Dice_Coef**	**Learning_rate**	**Loss**	**Val_Accuracy**	**Val_Dice_Coef**	**Val_Loss**
**1**	0.8755033612	0.1268693805	1.00E-04	0.8737280965	0.6669170267	0.01010781627	0.9898920655
**2**	0.9735860825	0.2358973771	1.00E-04	0.7641083598	0.4701127112	0.01231832802	0.9876815677
**3**	0.9821898937	0.3287226856	1.00E-04	0.6712055206	0.8782060742	0.04496941343	0.9550305009
**4**	0.9875227809	0.4348286986	1.00E-04	0.5652271509	0.9857260585	0.30371543774	0.6962844729
**5**	0.9892819524	0.5151044138	1.00E-04	0.4848868847	0.9895787239	0.40732169151	0.5926782489
**6**	0.9906331897	0.5828794837	1.00E-04	0.4168939292	0.9896375537	0.46581265338	0.5341873765
**7**	0.9924548268	0.6465564371	1.00E-05	0.3531814516	0.9925859571	0.62531971936	0.3746802509
**8**	0.9931911237	0.6752011776	1.00E-05	0.3244317772	0.9928584099	0.63849747181	0.6120834354
**9**	0.9935232997	0.6881361604	1.00E-05	0.3114761412	0.9929502012	0.64592719087	0.3540728986
**10**	0.9938025475	0.7000811696	1.00E-05	0.2995295227	0.9929558635	0.65453839398	0.3454615176
**11**	**0.9940174818**	0.7103929527	1.00E-05	0.2891717851	**0.9930544496**	0.65073972942	0.3492602408
**12**	0.9942512512	0.7180898786	1.00E-06	0.2814793289	0.9930226207	0.66768598564	0.3323140144
**13**	0.9942882657	0.7203966975	1.00E-06	0.2791729271	0.9930126071	0.66951233152	0.3304876983
**14**	0.9943166375	0.7217175364	1.00E-06	0.2778517604	0.9930288196	**0.67008185396**	**0.3299181163**
**15**	0.9943423271	0.7228963375	1.00E-06	0.2766736746	0.9930410981	0.67214852553	0.3278514745
**16**	0.9943641424	0.7244273428	1.00E-06	0.2755726573	0.9930515289	0.67235594974	0.3276440503
**17**	0.9943837528	0.7258619432	1.00E-06	0.2741380632	0.9930619597	0.67256343368	0.3274365664
**18**	0.9944029455	0.7272826437	1.00E-07	0.2727173563	0.9930723906	0.67277091746	0.3272290826
**19**	0.9944218397	0.7286458619	1.00E-07	0.2713541389	0.9930828214	0.67297840124	0.3270215988
**20**	0.9944404364	0.7299632437	1.00E-07	**0.2699230016**	0.9930932522	0.67318588529	0.3268141153
**21**	0.9944587941	**0.7312440872**	1.00E-08	0.2684845331	0.9931036837	0.67339336876	0.3266066313
**22**	0.9944770143	0.7324959039	1.00E-08	0.2670474648	0.9931141138	0.6736008525	0.3263991475
**23**	0.9944950938	0.7337275146	1.00E-08	0.2656123934	0.9931245446	0.6738083956	0.3261916044
**24**	0.9945130343	0.7349337936	**1.00E-09**	0.2641786332	0.9931349755	0.6740158794	0.3259841206
**25**	**0.9945308566**	**0.7361143827**	**1.00E-09**	**0.2627463342**	**0.9931454063**	**0.6742234225**	**0.3257765775**

**Table 8:** Evolution of segmentation performance metrics (IoU, Sensitivity, Specificity, and Boundary F1).

**Epoch**	**IoU (%)**	**Sensitivity (%)**	**Specificity (%)**	**Boundary F1 (%)**
**1**	0.5088723231	1.0107816270	99.8000000000	0.0107816270
**2**	0.6195887365	1.2318328020	99.7500000000	0.0123183280
**3**	2.3005177418	4.4969413430	99.6000000000	3.4969413430
**4**	17.8897305624	30.3715437740	99.2000000000	29.3715437740
**5**	25.5852609417	40.7321691510	99.1000000000	39.7321691510
**6**	30.3923838755	46.5812653380	99.0500000000	45.5812653380
**7**	45.5028384719	62.5319719360	99.2000000000	61.5319719360
**8**	46.9170643806	63.8497471810	99.2200000000	62.8497471810
**9**	47.6979332148	64.5927190870	99.2300000000	63.5927190870
**10**	48.6323579486	65.4538393980	99.2500000000	64.4538393980
**11**	48.1787415582	65.0739729420	99.2600000000	64.0739729420
**12**	50.1324601783	66.7685985640	99.2800000000	65.7685985640
**13**	50.3910478346	66.9512331520	99.2800000000	65.9512331520
**14**	50.4827263451	67.0081853960	99.2900000000	66.0081853960
**15**	50.8121934705	67.2148525530	99.2900000000	66.2148525530
**16**	50.8489502394	67.2355949740	99.3000000000	66.2355949740
**17**	50.8857670943	67.2563433680	99.3000000000	66.2563433680
**18**	50.9226440498	67.2770917460	99.3000000000	66.2770917460
**19**	50.9595811208	67.2978401240	**99.3100000000**	66.2978401240
**20**	50.9965783219	67.3185885290	99.3100000000	66.3185885290
**21**	51.0336356681	67.3393368760	99.3100000000	66.3393368760
**22**	51.0707531742	67.3600852500	99.3100000000	66.3600852500
**23**	51.1079308553	67.3808395600	99.3100000000	66.3808395600
**24**	51.1451687264	67.4015879400	99.3100000000	66.4015879400
**25**	**51.1824668028**	**67.4223422500**	99.3100000000	**66.4223422500**

**Table 9:** Segmentation performance metrics using Attention U-Net model.

**Epoch**	**Accuracy**	**Dice_Coef**	**Learning_rate**	**Loss**	**Val_Accuracy**	**Val_Dice_Coef**	**Val_Loss**
**1**	0.8450098634	0.1461230069	1.00E-04	0.8538768889	0.9822565317	0.0113776084	0.9886121753
**2**	0.9228816032	0.2260159254	1.00E-04	0.7739840746	0.9822412729	0.0006222074	0.9993770719
**3**	0.9464499965	0.2847048938	1.00E-04	0.7152951368	0.9822107553	0.0002038845	0.9997957349
**4**	0.9601147175	0.3449630439	1.00E-04	0.6550364494	0.9304416776	0.0867093205	0.9123817682
**5**	0.9696949728	0.4007241428	1.00E-04	0.5992760658	0.8841253519	0.1758239418	0.8224113584
**6**	0.9782437682	0.4773896933	1.00E-04	0.5226103067	0.9860045914	0.6070013046	0.3909461498
**7**	0.9836688638	0.5466272831	1.00E-04	0.4533727467	0.9854739308	0.5917394161	0.4053502679
**8**	0.9865750671	0.6018304825	1.00E-04	0.3981696963	0.9829621911	0.6078193784	0.3947688937
**9**	0.9898670316	0.6635155678	1.00E-05	0.3364844024	0.9903169274	0.7542915942	0.2479742914
**10**	0.9912266135	0.6875550747	1.00E-05	0.3124449253	0.9903558493	0.7274800539	0.2727259099
**11**	0.9919146299	0.7003290653	1.00E-05	0.2996711135	**0.9916796684**	0.7320810556	0.2679242793
**12**	0.9923691754	0.7121605873	1.00E-05	0.2877839204	0.9917556643	0.7236577868	0.2760605514
**13**	0.9929248095	0.7222942718	1.00E-06	0.2777053714	0.9916632175	0.7245507247	0.2755188644
**14**	**0.9932439327**	0.7303920984	1.00E-06	0.2696079016	0.9916355014	0.7073665261	0.2926014066
**15**	0.9933639765	0.7330074906	1.00E-06	0.2669925392	0.9917860031	0.7022882745	0.2974368632
**16**	0.9934318068	0.7367843527	1.00E-06	0.2632156487	0.9916603566	0.7005014725	0.2994989452
**17**	0.9934959413	0.7395004951	**1.00E-07**	0.2604995049	0.9916229248	0.6939265123	0.3060734868
**18**	0.9935479165	**0.7414526717**	1.00E-07	**0.2585473283**	0.9916111236	0.6872983457	0.3127016542
**19**	**0.9935889847**	**0.7435480958**	**1.00E-07**	**0.2564519042**	**0.9915511019**	**0.6760458725**	**0.3239541275**

**Table 10:** Training dynamics and convergence behavior of segmentation performance metrics.

**Epoch**	**IoU (%)**	**Sensitivity (%)**	**Specificity (%)**	**Boundary F1 (%)**
**1**	0.5720460143	1.1377608400	99.2200000000	0.1277608400
**2**	0.0311103596	0.0622207400	99.2200000000	0.0522207400
**3**	0.0101955156	0.0203884500	99.2200000000	0.0103884500
**4**	4.7457764389	8.6709320500	98.8000000000	7.6709320500
**5**	9.6320778154	17.5823941800	98.4000000000	16.5823941800
**6**	43.6151826297	60.7001304600	99.0000000000	59.7001304600
**7**	42.0415635024	59.1739416100	98.9500000000	58.1739416100
**8**	43.7118194426	60.7819378400	98.9000000000	59.7819378400
**9**	**60.5307419638**	**75.4291594200**	99.0300000000	**74.4291594200**
**10**	57.1206946295	72.7480053900	99.0400000000	71.7480053900
**11**	57.8473289893	73.2081055600	**99.1700000000**	72.2081055600
**12**	56.7321427068	72.3657786800	99.1700000000	71.3657786800
**13**	56.8532859811	72.4550724700	99.1600000000	71.4550724700
**14**	54.7320024051	70.7366526100	99.1600000000	69.7366526100
**15**	54.1189995155	70.2288274500	99.1700000000	69.2288274500
**16**	53.9082338094	70.0501472500	99.1600000000	69.0501472500
**17**	53.1506237839	69.3926512300	99.1600000000	68.3926512300
**18**	52.4096391164	68.7298345700	99.1600000000	67.7298345700
**19**	51.0897154286	67.6045872500	99.1500000000	66.6045872500

**Table 11:** Segmentation performance metrics using proposed SCSFE-DiagBT (Stacking segmentation model).

**Epoch**	**Accuracy**	**Dice_Coef**	**Learning_rate**	**Loss**	**Val_Accuracy**	**Val_Dice_Coef**	**Val_Loss**
**1**	0.9803817868	0.4159392715	1.00E-04	0.5824849015	0.9855237007	0.0012630396	0.9987368584
**2**	0.9881899357	0.5369921327	1.00E-04	0.4609683454	0.9854490757	0.0003369443	0.9996630549
**3**	0.9893646424	0.5984022021	1.00E-04	0.4007888734	0.9483339787	0.1604288227	0.8395712376
**4**	0.9907278419	0.6573613286	1.00E-04	0.3420932293	0.9896895885	0.6028084755	0.3971914351
**5**	0.9916724563	0.6969386935	1.00E-04	0.3022053547	0.9900008831	0.6466234326	0.3533766278
**6**	0.9924497008	0.7284687757	1.00E-04	0.2708572745	0.9905836582	0.5495477319	0.4504523277
**7**	0.9930924773	0.7537019849	1.00E-04	0.2453817725	0.9899800042	0.5445572734	0.4554426674
**8**	0.9939797521	0.7924262285	1.00E-05	0.2072347254	0.9931152463	0.7314459682	0.2685538828
**9**	0.9946555495	0.8168454174	1.00E-05	0.1830002517	0.9932975769	0.7426063418	0.2573936284
**10**	0.9949541688	0.8283029795	1.00E-05	0.1715873629	0.9933587909	0.7480530739	0.2519468963
**11**	**0.9952006347**	0.8378329277	1.00E-05	0.1620656103	0.9934301972	0.7507312298	0.2492686957
**12**	0.9954252243	0.8465353847	1.00E-05	0.1533658057	0.9935030341	0.7530965805	0.2469034344
**13**	0.9956419468	0.8549273014	1.00E-06	0.1449421048	0.9935606122	0.7580091357	0.2419908792
**14**	0.9956774116	0.8562839627	1.00E-06	0.1435866654	0.9935700893	0.7588572502	0.2411425114
**15**	0.9957040548	0.8573205471	1.00E-06	0.1425529569	0.9935827856	0.7593524458	0.2406473458
**16**	0.9957262873	0.8582319523	1.00E-06	0.1417680476	0.9935931568	0.7597651483	0.2402348517
**17**	0.9957445264	0.8589865021	1.00E-06	0.1410134979	**0.9936035271**	**0.7601777912**	**0.2398223286**
**18**	0.9957596768	0.8596625067	1.00E-07	0.1403374933	0.9936140175	0.7605902557	0.2394097443
**19**	0.9957717066	**0.8602327102**	1.00E-07	**0.1398101214**	0.9936244488	0.7608156204	0.2391843796
**20**	0.9957828522	0.8607365723	1.00E-07	0.1393291652	0.9936348796	0.7610233421	0.2389766579
**21**	0.9957937002	0.8611863856	1.00E-08	0.1388908921	0.9936453104	0.7612309451	0.2387690549
**22**	0.9958044882	0.8616352086	1.00E-08	0.1384539338	0.9936557412	0.7614385481	0.2385614519
**23**	0.9958156943	0.8621137142	1.00E-08	0.1380093482	0.9936661720	0.7616460915	0.2383539085
**24**	0.9958264823	0.8625609874	1**.00E-09**	0.1375661049	0.9936766028	0.7618535752	0.2381464248
**25**	**0.9958373303**	**0.8630497456**	**1.00E-09**	**0.1371241956**	**0.9936870337**	**0.7620611191**	**0.2379388809**

**Table 12:** Convergence analysis and performance progression of the proposed segmentation model.

**Epoch**	**IoU (%)**	**Sensitivity (%)**	**Specificity (%)**	**Boundary F1 (%)**
**1**	0.0632305412	0.1263039600	99.5500000000	0.1163039600
**2**	0.0168482645	0.0336944300	99.5400000000	0.0236944300
**3**	8.7175660485	16.0428822700	98.9500000000	15.0428822700
**4**	43.1418906247	60.2808475500	99.0000000000	59.2808475500
**5**	47.7679018324	64.6623432600	99.0500000000	63.6623432600
**6**	37.8900174928	54.9547731900	99.0600000000	53.9547731900
**7**	37.4175071093	54.4557273400	99.0500000000	53.4557273400
**8**	57.6865142829	73.1445968200	99.3100000000	72.1445968200
**9**	59.0647328573	74.2606341800	99.3300000000	73.2606341800
**10**	59.7864962327	74.8053073900	99.3400000000	73.8053073900
**11**	60.1453774635	75.0731229800	99.3400000000	74.0731229800
**12**	60.4674128161	75.3096580500	99.3500000000	74.3096580500
**13**	61.1598842455	75.8009135700	99.3500000000	74.8009135700
**14**	61.2862604638	75.8857250200	99.3500000000	74.8857250200
**15**	61.3603229084	75.9352445800	**99.3600000000**	74.9352445800
**16**	61.4234578881	75.9765148300	99.3600000000	74.9765148300
**17**	61.4866680876	76.0177791200	99.3600000000	75.0177791200
**18**	61.5499535328	76.0590255700	99.3600000000	75.0590255700
**19**	61.5847245086	76.0815620400	99.3600000000	75.0815620400
**20**	61.6166824787	76.1023342100	99.3600000000	75.1023342100
**21**	61.6486391034	76.1230945100	99.3600000000	75.1230945100
**22**	61.6805943932	76.1438548100	99.3600000000	75.1438548100
**23**	61.7125483586	76.1646091500	99.3600000000	75.1646091500
**24**	61.7445009999	76.1853575200	99.3600000000	75.1853575200
**25**	**61.7764523276**	**76.2061119100**	99.3600000000	**75.2061119100**

**Table 13:** Comparaison of classification performance metrics.

Refs/Year	Meningioma				Glioma				Pituitary				No Tumor			
	**Accuracy**	**Precision**	**Recall**	**F1-Score**	**Accuracy**	**Precision**	**Recall**	**F1-Score**	**Accuracy**	**Precision**	**Recall**	**F1-Score**	**Accuracy**	**Precision**	**Recall**	**F1-Score**
[6] / 2025	0.990	0.980	0.980	-	0.980	0.980	0.970	-	0.990	0.990	0.980	-	-	-	-	-
[12] / 2025	0.962	0.930	0.920	0.923	0.968	0.970	0.960	0.963	**1.000**	**1.000**	**1.000**	**1.000**	0.986	0.974	0.982	0.976
[44] / 2025	0.992	0.980	0.985	0.983	0.993	0.990	0.995	0.990	0.994	0.996	0.986	0.989	-	-	-	-
[9] / 2025	-	0.910	0.920	0.920	-	0.940	0.950	0..940		0.960	0.960	0.960	-	-	-	-
[45] / 2025	-	0.863	0.919	-	-	0.874	0.815	-	-	0.976	0.835	-	-	-	-	-
[46] / 2025	0.993	-	0.990	-	0.993	-	0.980	-	0.991	-	0.990	-	-	-	-	-
SCSFE-DiagBT	**0.995**	**0.997**	**1.000**	**0.998**	**1.000**	**1.000**	**1.000**	**1.000**	**1.000**	**1.000**	**1.000**	**1.000**	**1.000**	**0.999**	**1.000**	**0.999**

**Table 14:** Comparative analysis of classification and segmentation performance metrics for brain tumor diagnosis.

**Refs/Year**	**Model**	**Dataset**	**Accuracy**
**[** **47** **] / 2020**	Customized ResNet50 model	253 MRIs (Kaggle dataset)	97.01%
**[** **48** **] / 2020**	AlexNet, VGG16, AlexNet+VGG16 with hyper-column techniques and SVM	253 MRIs from Kaggle	AlexNet: 92.47% VGG16: 90.32% AlexNet+VGG16: 96.77%
**[** **49** **] / 2021**	Multi-scale CNN	Figshare	97.3%
**[** **50** **] / 2021**	Improved ResNet50 Model	551 healthy and diseased images Kaggle and http://radiopaedia.org/	98.59%
**[** **51** **] / 2021**	Feature level ensemble of 3 CNNs with PCA feature reduction	Figshare	98.37%
**[** **52** **] / 2022**	Ensemble of vision transformers	Fighshare	98.7%
**[** **53** **] / 2022**	Custom deep neural network model with less hyper-parameters	Figshare	99.18%
**[** **54** **] / 2022**	3D U-Net and 16-layer CNN	i. BRATS2020 for segmentationmodel ii. Kaggle dataset 3264 images for classification model	99.06% 90%
**[** **55** **] / 2025**	IV3TM	Figshare,	98.02%
**[** **56** **] / 2023**	ResNet with hyper-parameter optimization	Figshare	98.6%
**[57] /2024**	CSA-MLP	Kaggle	98.67%
**[** **58** **] / 2024**	MC-SVM	BRATS	99.06%
**[** **12** **] / 2025**	Deep CNN	Figshare	97.27%
**[** **44** **] / 2025**	BO-DenseXGB	Figshare	99.02%
**[** **45** **] / 2025**	Enhanced Spatial Attention (ESA)	Figshare	92%
**[** **59** **] / 2025**	CNNL Ensemble voting classifier	Dataset from Tianjin Medical University, China	99.69%
**[** **8** **] / 2025**	Explainable deep stacking ensemble model	BraTS, Msoud, Br35H, and SARTAJ	99.32 ± 0.46 98.09 ± 0.21 98.16 ± 0.35 98.14 ± 0.52
**[** **60** **] / 2025**	EWHO-SqueezeNet	BRATS Figshare	92.99% 93.20%
**Proposed system 2025**	**SCSFE-DiagBT**	**Kaggle Brain tumor MRI dataset** **Figshare** **BRATS**	**99.95%** **99.91** **99.97**

## Data Availability

The datasets created and analyzed during this study are publicly available at the following website: https://www.kaggle.com/datasets/nikhilroxtomar/brain-tumor-segmentation.

## Footnotes

1
https://www.kaggle.com/datasets/nikhilroxtomar/brain-tumor-segmentation

2
https://universe.roboflow.com/ali-rostami/labeled-mri-brain-tumor-dataset/data set/1

## LIST OF ABBREVIATIONS

SCSFE-DiagBT: Stacked Classification and Segmentation Model with Feature Extraction for Brain Tumor Diagnosis
AI: Artificial Intelligence
MRI: Magnetic Resonance Imaging
EEG: Electroencephalography
CNN: Convolutional Neural Network
CLAHE: Contrast Limited Adaptive Histogram Equalization
PCA: Principal Component Analysis
SMOTE: Synthetic Minority Over-sampling Technique
Grad-CAM: Gradient-weighted Class Activation Mapping
VGG-n: Visual Geometry Group n-layer Network
YOLO: You Only Look Once
U-Net: U-shaped Convolutional Network
ResNet: Residual Neural Network
Xception: Extreme Inception
ROC-AUC: Receiver Operating Characteristic – Area Under the Curve
